# Prognostic impact of peak oxygen consumption in heart failure: A systematic review and meta‐analysis

**DOI:** 10.1002/ehf2.15391

**Published:** 2025-08-12

**Authors:** Konstantinos Prokopidis, Krzysztof Irlik, Julia Piaśnik, Zuzanna Michalska, Mirela Hendel, Katarzyna Nabrdalik, Gregory Y.H. Lip

**Affiliations:** ^1^ Department of Musculoskeletal and Ageing Science, Institute of Life Course and Medical Sciences University of Liverpool Liverpool UK; ^2^ Liverpool Centre for Cardiovascular Science University of Liverpool, Liverpool John Moores University and Liverpool Heart and Chest Hospital Liverpool UK; ^3^ Students' Scientific Association by the Department of Internal Medicine, Diabetology and Nephrology, Faculty of Medical Sciences in Zabrze Medical University of Silesia Katowice Poland; ^4^ Doctoral School, Department of Internal Medicine, Diabetology and Nephrology, Faculty of Medical Sciences in Zabrze Medical University of Silesia Katowice Poland; ^5^ Department of Internal Medicine, Faculty of Medical Sciences University of Warmia and Mazury in Olsztyn Olsztyn Poland; ^6^ Department of Internal Medicine, Diabetology and Nephrology, Faculty of Medical Sciences in Zabrze Medical University of Silesia Katowice Poland; ^7^ Danish Center for Health Services Research, Department of Clinical Medicine Aalborg University Aalborg Denmark

**Keywords:** heart failure, hospitalization, mortality, peak oxygen uptake, VO_2_peak

## Abstract

**Background and Aims:**

Heart failure (HF) is a multifactorial disease for which peak oxygen uptake (VO_2_peak) may potentially be a prognostic marker of adverse clinical outcomes. This systematic review and meta‐analysis aimed to assess published data on the prognostic impact of VO_2_peak in HF.

**Methods:**

A literature search of observational studies was conducted through PubMed, Scopus, Web of Science and Cochrane Library from inception until January 2025. A meta‐analysis was conducted using the random‐effects inverse‐variance model through hazard ratios (HRs). Increased heterogeneity among studies was evaluated through meta‐regressions and publication bias via Egger's test.

**Results:**

Sixty‐four studies were included in this systematic review and meta‐analysis. Per 1 mL/kg/min increase in VO_2_peak, all‐cause mortality [HR: 0.86, 95% confidence interval (CI) 0.82–0.90, *I* ^2^ = 85%, *P* < 0.01] and incident ventricular assist device, transplant and all‐cause mortality (HR: 0.84, 95% CI 0.79–0.89, *I* ^2^ = 33%, *P* < 0.01) were significantly reduced, but statistical significance of VO_2_peak with cardiovascular mortality was not observed (HR: 0.92, 95% CI 0.82–1.02, *I* ^2^ = 0%, *P* = 0.12) using adjusted models. Variance among studies was detected based on age, sex, body mass index, left ventricular ejection fraction, atrial fibrillation, hypertension, chronic kidney disease, diabetes and treatment. A significant risk of publication bias was evident.

**Conclusions:**

VO_2_peak is a prognostic marker for multiple causes of mortality and hospitalization in patients with HF, which may promote further insights into patient risk stratification for adverse events and targeted management.

## Graphical abstract

Based on 64 studies, each 1 mL/kg/min increase in VO2peak significantly reduced all‐cause mortality (HR: 0.86, 95% CI 0.82–0.90) and combined outcomes of ventricular assist device, transplant and all‐cause mortality (HR: 0.84, 95% CI 0.79–0.89) in patients with heart failure, though no significant association was observed with cardiovascular mortality (HR: 0.92, 95% CI 0.82–1.02). Variance was influenced by age, sex and comorbidities. VO2peak may serve as a prognostic marker for mortality and hospitalisation in patients with heart failure.

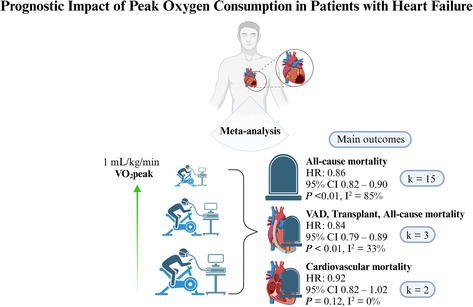

## Introduction

Heart failure (HF) represents a clinical condition characterized by structural and/or functional cardiac impairment that results in increased intracardiac pressures and/or inadequate cardiac output at rest or during exercise. A multitude of factors contribute to the heterogeneous nature of HF, and understanding its prognostic markers is critical for its effective management. Among these markers, peak oxygen uptake (VO_2_peak) has emerged as a pivotal physiological parameter with profound implications for risk stratification in patients with HF.^1^

VO_2_peak refers to the highest rate of oxygen consumption attained during an exercise test, reflecting an individual's aerobic capacity under specific testing conditions, and serves as a reflection of cardiovascular (CV) and pulmonary function.^2^ More specifically, the compromised cardiac function in HF may directly influence the ability of the CV system to deliver oxygen to peripheral tissues during exercise.^3^ Low VO_2_peak has been consistently linked to increased all‐cause mortality, as well as CV mortality and HF hospitalization.^4^, ^5^, ^6^ These results advocate for rehabilitation strategies that target VO_2_ improvement, leading to better clinical outcomes.^7^ Recent advances in cardiopulmonary exercise testing (CPET) emphasize using predicted percentage values for VO_2_peak and VE/VCO_2_ slope to improve comparisons across populations. Currently, non‐invasive methods allow assessment of cardiac output and oxygen extraction, which possess a growing role in personalized HF management.^8^ Therefore, the prognostic significance of VO_2_peak extends beyond its role as a marker of functional capacity, serving as a clinical tool for risk stratification, aiding clinicians in identifying patients at increased risk for adverse HF‐related events. In particular, risk‐stratifying individuals based on their exercise capacity may enable healthcare professionals to implement targeted treatment strategies in order to optimize outcomes for the enhancement of overall patient care and rehabilitation.

In this systematic review and meta‐analysis, we attempt to assess evidence for the prognostic impact of VO_2_peak in HF, aiming to raise awareness regarding its integration into clinical practice to facilitate HF management.

## Methods

This systematic review and meta‐analysis was conducted according to the Preferred Reporting Items for Systematic Reviews and Meta‐Analyses (PRISMA) guidelines (Data S1).^9^ The protocol has been registered in the International Prospective Register of Systematic Reviews (PROSPERO) (CRD42024519903).

### Search strategy

Two independent reviewers searched PubMed, Scopus, Web of Science and Cochrane Library from 1964 until January 2025. The full search strategies employed are shown in *Table* S1. Discrepancies in the literature search process were resolved by a third investigator.

### Inclusion and exclusion criteria

Studies were included based on the following criteria: (i) prospective cohort studies using hazard ratio (HR)/risk ratio (RR) to assess the prognostic impact of VO_2_peak on mortality and (ii) patients irrespective of HF phenotype (e.g., with reduced or preserved ejection fraction) aged 18 years and above. Published articles were excluded if they were (i) reviews, letters, in vivo or in vitro experiments, or commentaries and (ii) not published as a full text and in English.

### Data extraction and risk of bias

Two authors extracted data independently, which included the name of the first author, date of publication, country of origin, population of cohort, sample size, age, body mass index (BMI), left ventricular ejection fraction (LVEF), New York Heart Association (NYHA) functional classification, hypertension, chronic kidney disease, atrial fibrillation and diabetes prevalence, sex of participants, type of HF, outcomes of interest and follow‐up duration. Disagreements between authors were resolved by an independent reviewer. The quality of the included studies was evaluated using the Newcastle–Ottawa Scale (NOS) by three investigators.^10^ The NOS assigns a maximum of 9 points based on three quality parameters: selection, comparability and outcome, and risk of bias was categorized as high (≤5 points), moderate (6–7) or low (8–9).^11^

### Data synthesis

In this meta‐analysis, statistical significance was assessed using the random‐effects model and inverse‐variance method in outcomes with two or more studies using HR or RR. To investigate more reliable and homogenous results, we performed separate analyses based on included studies utilizing adjusted and unadjusted models. The method ‘standard deviation (SD) = width of IQR/1.35’ was employed to approximately calculate the missing SDs when studies reported the inter‐quartile range (IQR). Where 95% confidence intervals (CIs) were available, SDs were obtained using the equation ‘SD = √*N* × (upper limit of CI − lower limit of CI)/3.92’.^12^ In addition, statistical heterogeneity of outcome measurements between different studies was assessed using the overlap of their 95% CI and was expressed as measurements of Cochran's *Q* (chi‐squared test) and *I* ^2^. The classification of data as having low, moderate and high heterogeneity was based on *I* ^2^ from 30% to 49%, 50% to 74%, and 75% and above, respectively.^13^ Sensitivity analysis was employed to evaluate the robustness of reported statistical results by discounting the effects of studies with increased risk of bias. Publication bias was assessed by visually inspecting funnel plots and Egger's test in analyses including ≥10 studies, for which a *P* value <0.05 was an indicator of potential publication bias.^14^ If publication bias was detected, we employed the trim‐and‐fill method, an approach that operates under the assumption that effect sizes from all studies follow a normal distribution around the centre of a funnel plot. If asymmetries are present, the method adjusts for the potential influence of unpublished (trimmed) studies.^15^ Egger's test and the trim‐and‐fill method were conducted in R Version 4.3.3. The meta‐analysis was synthesized using Review Manager (RevMan 5.4.1) software. In case of increased heterogeneity (*I* ^2^ ≥ 50%) for studies using adjusted models, a meta‐regression was conducted to account for age, sex, BMI, LVEF, atrial fibrillation, hypertension, chronic kidney disease and diabetes, when observations were sufficient, using Stata/MP 13.0. A *P* value of <0.05 was considered statistically significant.

### Outcomes of interest

The following outcomes were used to explore the prognostic impact of VO_2_peak in patients with HF: all‐cause mortality; CV mortality; ventricular assist device (VAD) implantation, heart transplant and all‐cause mortality; all‐cause mortality or HF rehospitalization; all‐cause mortality or HF hospitalization; CV mortality or HF rehospitalization; VAD implantation, heart transplant, CV mortality or HF hospitalization; heart transplant and all‐cause mortality; heart transplant and CV mortality; and HF hospitalization. Units of change of VO_2_peak used in the analysis were 1 mL/kg/min, ≤14 mL/kg/min and VO_2_peak < 50% of predicted value. The duration of the measured outcomes varied between 6 months and 10 years.

## Results

**Figure 1:**
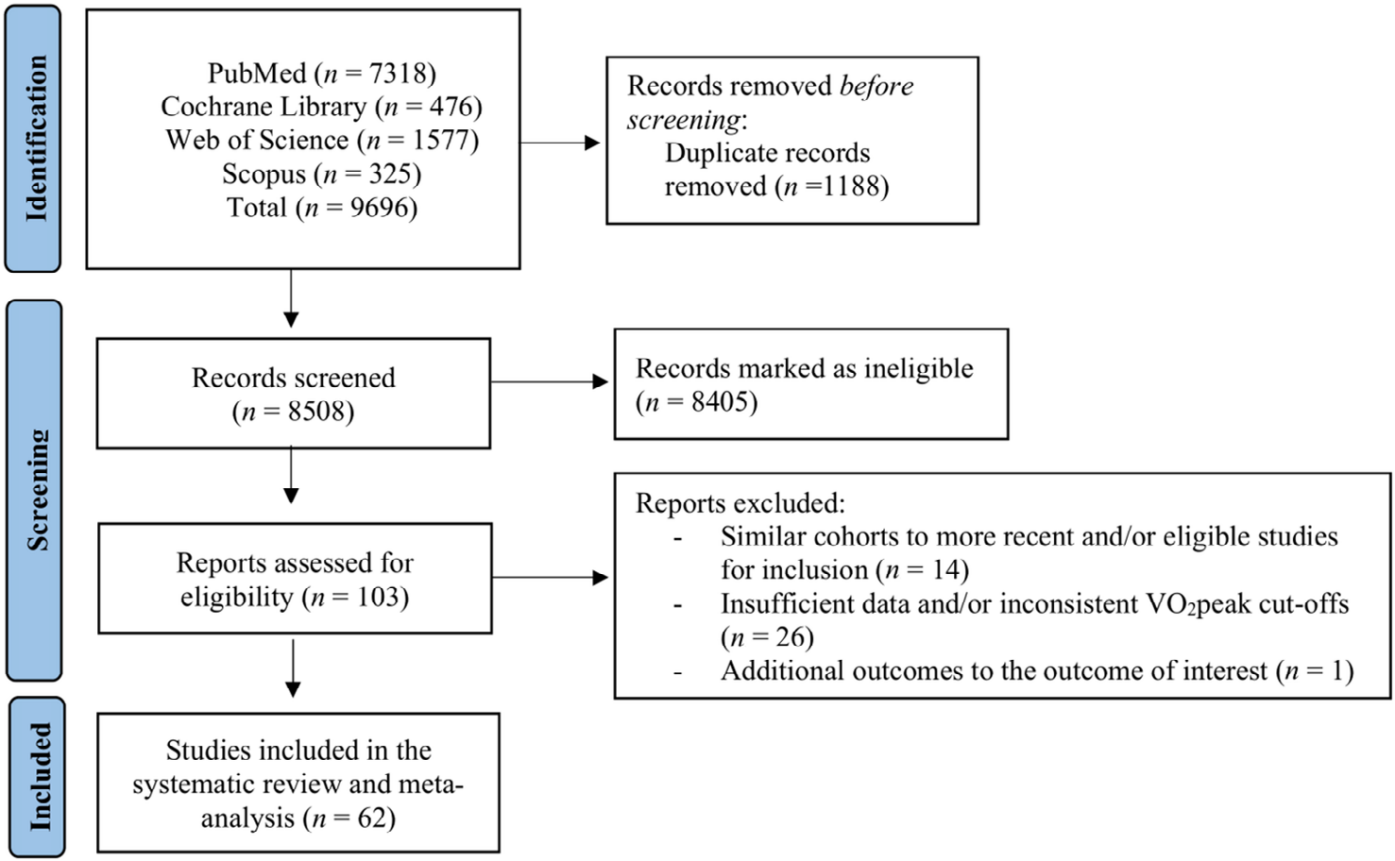
The Preferred Reporting Items for Systematic Reviews and Meta‐Analyses flow chart of studies included in the meta‐analysis.

The initial literature search yielded 9696 publications. Following the exclusion of duplicates and abstracts, 105 full texts were assessed for eligibility. From these, 26 studies had insufficient data based on 95% CI in HRs and inconsistent VO_2_peak cut‐offs when evaluating HRs. Additionally, 14 studies consisted of identical cohorts to studies that were deemed as eligible for inclusion in this study, while one study had additional outcomes to our outcome of interest for investigation. Therefore, of the 105 studies, 64 studies were included in this systematic review and meta‐analysis. Flow chart of the article selection is presented in *Figure* *1*.

Overall, 28 studies evaluated the prognostic impact of VO_2_peak on all‐cause mortality; 6 studies on CV mortality; 5 studies on VAD, transplant and all‐cause mortality; 2 studies on all‐cause mortality and HF rehospitalization; 3 studies on VAD, transplant, CV mortality and HF hospitalization; 6 studies on all‐cause mortality and HF hospitalization; 6 studies on CV mortality and/or HF rehospitalization; 8 studies on transplant and all‐cause mortality; 3 studies on transplant and CV mortality; and 3 studies on HF hospitalization. Details of the included studies are presented in *Table* *1*. References for included studies for each outcome are provided in *Table* S2.

**Table 1:** Study and participant characteristics of the included studies evaluating the impact of VO_2_peak on the outcomes of interest.

Study, year	Sample size (M/F)	Age	BMI	LVEF (%)	VO_2_peak at baseline	Duration of measured outcome	Method of VO_2_peak assessment	VO_2_peak Assessment Modality	Confounders adjusted for
Al‐Najjar 2012^16^	411 (335/76)	65.7 ± 10.8	25.5 ± 5.4	‐	19.8 ± 6.0	1 year	The average VO_2_reading over the last 30 s of exercise	Treadmill	Age and beta‐blocker treatment
Baldi 2019^17^	74 (58/16)	71.6 ± 8.3	‐	33.2 ± 6.8	10.5 ± 2.7	1 year/median 416.0 days	The highest VO_2_ normalized to body weight (VO_2_, mL/kg/min) for a given 15 or 20 s interval	Upright cycle ergometer	AF
Bras 2023^18^	487 (385/102)	56 ± 13	27.1 ± 4.3	29.8 ± 7.9	18.3 ± 5.9	3 years	Modified Bruce protocol: The highest 30 s average achieved during exercise and was normalized for body mass	Treadmill	BMI, LVEF, eGFR, VE/VCO_2_ slope, O_2_ pulse, circulatory power, ventilatory power, cardiorespiratory optimal point, partial pressure of end‐tidal carbon dioxide at rest and partial pressure of end‐tidal carbon dioxide at anaerobic threshold
Chen 2023^19^	377 (294/83)	59 ± 12.65	25.93 ± 4.91	28.67 ± 8.2	16.33 ± 4.76	Median follow‐up period: 3.3 years	The highest 30 s average value obtained during exercise with a respiratory exchange ratio of at least 1.05	Treadmill or upright cycle ergometer	Age, BNP, Na, DM, hyperlipidaemia, stroke, AF, ICD, eGFR and VE/VCO_2_ slope
Chen 2024^20^	247 (194/53)	59.3 ± 11.9	26.0 ± 4.2	29.3 ± 7.5	15.93 ± 4.1	Median follow‐up period: 3.3 years	Modified Bruce or Cornell protocol	Treadmill or upright cycle ergometer	Cardiac rehabilitation, Luria–Nebraska Neuropsychological Battery‐screening test, sex, BMI, ischaemic cardiomyopathy, hypertension, DM, AF, mean blood pressure, heart rate, LVEF, serum sodium, Hb, beta‐blockers, ACEI/ARB/ARNI, Beck Anxiety Inventory, Beck Depression Inventory‐II, old stroke, eGFR and albumin
Corrà 2020^21^	744 (693/51)	60 ± 11	‐	28 ± 8	14.7 ± 4.3	1 year	Mean VO_2_ during the last 60 s of the test	Upright cycle ergometer	‐
Cunha 2023^22^	250 (188/62)	58.4 ± 11.6	27.1 ± 4.6	34 ± 9.5	18.3 ± 6.2	2 years	Bruce, modified Bruce or modified ramp protocols	Treadmill	LVEF, VE/VCO_2_ slope, NT‐proBNP and loop gain
Czubaszewski 2018^23^	132 (132/0)	‐	‐	‐	‐	2 years/27 ± 13 months	Mean value from the last 20–30 s of the test	Treadmill	‐
de Groote 2004^24^	407 (‐/‐)	57 ± 11	‐	33 ± 13	15.2 ± 4.8	Median follow‐up: 787 days	Not reported	Check	BNP, LAD, aldosterone and creatinine
Doehner 2005^25^	105 (105/0)	62 ± 1	24.7 ± 0.4	‐	18.2 ± 0.7	4 years	Modified Bruce protocol	Treadmill	‐
Ehrman 2018^26^	1085 (728/357)	54 ± 13	30 ± 7	22 ± 9	15.5 ± 4.9	Men: 5.7 (2.1–9.0) Women: 7.2 (3.5–10.8)	The highest interval value during the final minute of exercise based on 15 s averaged data	Treadmill	Age
Badr Eslam 2022^27^	78 (68/10)	78 ± 6	25.5 ± 3.1	52 ± 11	15.0 ± 4.6	9 ± 3 months	The highest 30 s average of oxygen uptake in the last minute of exercise	Upright cycle ergometer	‐
Gitt 2002^28^	223 (192/31)	62.9 ± 10.7	26.5 ± 3.8	28.7 ± 8.1	15.8 ± 5.3	6 months	Highest VO_2_ achieved during exercise	Upright cycle ergometer	Age, sex, LVEF and NYHA
Goda 2011^29^	715 (464/251)	53.6 ± 11.6	27.5 ± 5.6	21.6 ± 7	13.6 ± 4.6	2 years/a follow‐up of 962 ± 912 days	Modified Naughton protocol	Treadmill	NYHA class, LVEF, BMI, beta‐blockers, rest SBP, RHR, loop diuretic dose equivalent, Na, lymphocytes percentage, presence of IVCD, uric acid, Hb, total cholesterol, statin and spironolactone
Gordon 2023^30^	2074 (1494/580)	‐	‐	‐	‐	30 months	Modified Naughton protocol	Treadmill	Age, race, sex, NYHA class, LVEF, history of MI, ischaemic vs. non‐ischaemic HF aetiology, beta‐blocker use, ACEI/ARB use, prior revascularization and VE/VCO_2_ slope
Guazzi 2009^31^	376 (278/98)	59.9 ± 11.9	27.6 ± 5.2	33.1 ± 12.1	5.2 ± 4.9	4 years	The highest 30 s averaged samples obtained during exercise	Treadmill or cycle ergometer	‐
Hansen 2001^32^	311 (261/50)	54 ± 10	‐	24 ± 12	14.7 ± 5.5	2 years/a mean follow‐up period of 512 ± 314 days	Not reported	Bicycle ergometer	Restrictive filling, AF vs. non‐restrictive filling pattern and LVED
Hoyer 2008^33^	200 (87/113)	53 ± 8.2	‐	23.3 ± 6.7	14.47 ± 4.48	Median: 31 months (range: 0.4–36 months)	Not reported	Not specified	NYHA class, LVEF, short‐term beat decay and systolic arterial pressure
Hsich 2007^34^	2105 (1580/525)	‐	‐	‐	‐	5 years	Naughton protocol	Not specified	Age, black race, LVEF, BMI, insulin‐treated DM, hypertension, current smokers, CAD, previous MI, previous coronary bypass, previous coronary angioplasty, pacemaker, ICD, AF, PVD, carotid artery disease, COPD, asthma, previous stroke, previous transient ischaemic attack, medication use [ACEI, ARB, amiodarone, beta‐blocker, calcium channel blockers, aspirin, statin, spironolactone (potassium‐sparing diuretics), digoxin (digitalis), loop diuretic and thiazide diuretic], RHR, SBP at rest, DBP at rest, peak RER and severe ventricular ectopy during recovery
Hsu 2019^35^	202 (58/144)	‐	‐	‐	‐	5 years	ACSM guidelines	Upright cycle ergometer	Age, sex, LVEF and LVESD
Ingle 2012^36^	411 (333/78)	64 ± 12	27.8 ± 5.3	‐	22.3 ± 8.1	Median: 8.7 ± 2.3 years	Mean VO_2_ in the final 30 s of exercise	Treadmill	‐
Jankowska^37^ 2007	216 (188/28)	60 ± 11	26.5 ± 4.3	31 ± 8	14.8 ± 4.9	3 years	Bruce protocol (average of the last 30 s of exercise)	Treadmill	Age, NYHA class, LVEF and VE‐VCO_2_‐all
Jorde 2007^38^	60 (44/16)	‐	‐	26.1 ± 1.2	15.0 ± 0.7	19 ± 4 months	Naughton protocol	Treadmill	Age, sex, LVEF, NYHA class and delta oxidized low‐density lipoprotein
Kallistratos 2008^39^	149 (122/27)	59 ± 13	‐	33.6 ± 9.3	17.6 ± 5	30 ± 10 months	Dargie protocol: The highest value in the terminal phase of exercise	Treadmill	Age and gender
Koerber 2020^40^	22 (17/5)	‐	29 ± 4	26 ± 13	11.9 ± 2.4	1 year	Naughton protocol	Recumbent bicycle ergometer	Peak CO and exercise‐induced hypoxaemia after adjusting for peak VO_2_ and peak CO
Koike 2000^41^	249 (151/98)	55.8 ± 13.3	‐	‐	23.2 ± 6.9	10 years	Mean VO_2_ in the final 30 s of exercise	Upright cycle ergometer	‐
Lala 2021^42^	237 (171/66)	60.4 ± 11.4	30.8 ± 6.8	29 ± 8	14.0 ± 4.3	1 year	Modified Naughton protocol	Treadmill	‐
Lamblin 2005^43^	545 (449/96)	‐	‐	‐	‐	3 years	Not reported	Not specified	Age, gender, BMI, NYHA class, LVEF, RVEF, BNP, DM, hypertension, hs‐CRP and ischaemic aetiology
Lee 2012^44^	131 (86/45)	59 ± 14	‐	‐	‐	1.2 ± 0.7 years	(1) The level of VO_2_ increased <2 mL/kg/min over at least 2 min; (2) heart rate exceeded 85% of its predicted maximal value; (3) respiratory exchange ratio exceeded 1.15; or (4) some other symptoms existed, based on ACSM guidelines	Upright cycle ergometer	DM, eGFR, Hb, oxygen uptake efficiency slope, VE/VCO_2_ slope and peak cardiac index
Li 2024^45^	154 (88/66)	57 ± 12.4	29.9 ± 7.4	66 ± 12.4	14.8 ± 3.72	Median: 5.6 years	Incremental exercise ramp protocol of 5–25 W/min	Upright cycle ergometer	‐
Magri 2015^46^	151 (134/17)	62 ± 11	27 ± 3	34 ± 8	16.1 ± 5.1	995 days	Ramp protocol	Upright cycle ergometer	‐
Magri 2020^47^	5711 (4764/947)	63 ± 13	27 ± 4	31 ± 6	11.4 ± 3.8	5 years	Sex‐, age‐ and weight‐adjusted Hansen/Wasserman equations	Upright cycle ergometer or treadmill	‐
Malhotra 2016^48^	103 (64/39)	61 ± 12	30.6 ± 6.8	‐	‐	4 years	The highest O_2_ uptake, averaged over 30 s, during the last minute of symptom‐limited exercise	Upright cycle ergometer	‐
Meyer 2001^49^	244 (207/37)	54 ± 11	‐	22 ± 10	14.6 ± 5.2	1 year (23 ± 16 months)	Modified Bruce protocol	Semi‐recumbent bicycle ergometer	LVEF, NYHA and maximal inspiratory pressure
Murata 2019^50^	58 (18/40)	83 ± 5	22.1 ± 3.6	62.0 ± 10.5	11.3 ± 3.3	19 ± 9 months	VO_2_ at peak delta VO_2_/delta work rate during exercise	Upright cycle ergometer	Age, sex, Hb and eGFR
Myers 2009^51^	847 (602/245)	57 ± 14	28.4 ± 5.9	32.1 ± 14.1	15.1 ± 4.9	3 years	The highest averaged VO_2_ samples obtained during the exercise test	Treadmill	‐
Myers 2021^52^	596 (2810/1245)	53.1 ± 15.3	28.0 ± 5.7	‐	19.2 ± 8.9		Averaging readings during the final 30–60 s of the cardiopulmonary exercise testing	Treadmill	ppVO_2_ FRIEND and ppVO_2_ Wasserman/Hansen
Nadruz 2017^4^	969 (654/315)	56 ± 14	28.6 ± 6.1	42 ± 7	13.3 ± 6.0	Median: 4.2 years	The highest 10 s averaged VO_2_ during the last stage of the symptom‐limited exercise test	Upright cycle ergometer or treadmill	Age, sex, EF, RHR, rest SBP and CAD
Nakanishi 2014^53^	283 (229/54)	61.8 ± 13.5	21.9 ± 3.7	26.3 ± 8.0	17.0 ± 4.4	4 years (median: 47 months)	The higher value normalized to body weight (mL/kg/min) of either the greatest VO_2_ during exercise (smoothed after a 5‐point moving average) or the average VO_2_ of the last 3 data points (18 s) before termination of exercise	Cycle ergometer	LVEF, BNP, LVDd, LVDs, LAD, creatinine, Hb, Na and VE/VCO_2_ slope
Nakanishi 2022^54^	772 (584/188)	62.2 ± 14.9	22.5 ± 3.8	30.8 ± 13.1	‐	Median: 4.6 years	The greatest VO_2_ during exercise or the average VO_2_ of the last 3 data points (18 s) before termination of exercise	Cycle ergometer	Age, sex, LVEF, BNP (per 10 pg/mL), LAD, LVDd, DM, AF, ICD/CRT, prior HF hospitalization, creatinine, Hb, VE/VCO_2_ slope and comprehensive cardiac rehabilitation completion
O'Neill 2005^55^	2104 (1578/526)	53.7 ± 10.8	‐	19 ± 7	16.6 ± 5.1	Median follow‐up: 3.5 years	Naughton protocol	Treadmill	Age, black race, LVEF, BMI, insulin‐treated DM, hypertension, current smokers, CAD, previous MI, previous coronary bypass, previous coronary angioplasty, pacemaker, ICD, AF, PVD, carotid artery disease, COPD, asthma, previous stroke, previous transient ischaemic attack, medication use [ACEI, ARB, amiodarone, beta‐blocker, calcium channel blockers, aspirin, statin, spironolactone (potassium‐sparing diuretics), digoxin (digitalis), loop diuretic and thiazide diuretic], RHR, SBP at rest, DBP at rest, peak RER and severe ventricular ectopy during recovery
Opasich 2001^56^	315 (274/41)	53 ± 9	‐	26 ± 8	14·6 ± 4·4	387 days	Not reported	Bicycle ergometer	Walking test distance
Piepoli 2016^57^	4623 (3828/795)	61.6 ± 12.6	26.2 ± 3.6	‐	‐	1000 person‐years	Modified Bruce protocol	Upright cycle ergometer or treadmill	‐
Pugliese 2019^58^	159 (130/29)	62.7 ± 10.5	26.67 ± 1.12	30 ± 6	16.7 ± 4.9	5 years	Ramp protocol: Predicted based on age, height, weight and clinical history	Semi‐recumbent bicycle ergometer	‐
Pugliese 2021^59^	393 (223/170)	68 ± 13	‐	‐	‐	22.9 months	The highest averaged 30 s VO_2_ during exercise	Semi‐recumbent bicycle ergometer	NT‐proBNP, echocardiographic epicardial adipose tissue, peak tricuspid annular plane systolic excursion/systolic pulmonary artery pressure, ΔB‐lines and VE/CO_2_ slope
Romuk 2020^60^	741 (636/105)	53.7 ± 8.2	26.37 ± 4.32	24.7 ± 7.4	14.55 ± 4.38	1 year	Modified Bruce protocol	Treadmill	BMI, NYHA class, LVEF, ceruloplasmin, NT‐proBNP, duration of symptoms before inclusion increase, Na, creatinine clearance, albumin, HDL cholesterol, fibrinogen, uric acid, bilirubin, alkaline phosphatase, gamma‐glutamyl transpeptidase, type 2 diabetes, ICD, ACEI or/and ARB, loop diuretics, thiazide diuretics, statins and digitalis
Saitoh 2016^61^	130 (105/25)	66.3 ± 11.5	28.4 ± 4.6	34.3 ± 10.7	17.9 ± 6.7	2 years	Modified Naughton protocol	Upright cycle ergometer	LVEF and Mini Nutritional Assessment—Short Form score
Sato 2015^62^	598 (476/122)	61.4 ± 14.3	23.4 ± 4.0	45.7 ± 14.5	16.3 ± 5.2	Median: 782 days	The average VO_2_ of the last 30 s of exercise	Upright cycle ergometer	Age, sex, BMI, LVEF, LAD, BNP, AF, eGFR, Na, Hb, deceleration time, RHR, rest SBP, blood urea nitrogen and VE/VCO_2_ slope
Scardovi 2012^63^	370 (107/263)	74.3 ± 5.2	25.4 ± 3.4	41.7 ± 11.9	11.93 ± 3.05	1 year	The highest averaged 10 s interval samples obtained during the exercise test	Upright cycle ergometer	‐
Shafiq 2016^64^	173 (96/77)	54 ± 14	32 ± 9	56 ± 5	16.9 ± 6.3	Median follow‐up: 5.2 years	Not reported	Treadmill	Age, gender and beta‐blocker therapy
Shen 2016^65^	129 (113/16)	59.1 ± 11.4	24.7 ± 3.7	38 ± 9	14.0 ± 3.9	6 years	Modified Ramp10 protocol	Upright cycle ergometer	Age, gender, BMI, RHR and LVMI
Shibata 2018^66^	94 (60/34)	68.0 ± 14.5	22.2 ± 4.0	38.4 ± 14.8	‐	6 months	The highest VO_2_ value achieved at peak exercise	Upright cycle ergometer	‐
Silverii 2023^67^	75 (68/7)	79.3 ± 6.0	‐	55 ± 10.6	14.1 ± 4.1	2 years	The average O_2_ uptake measured over the last 30 s of exercise	Bicycle ergometer	‐
Stolker 2006^68^	221 (150/71)	49 ± 12	29.3 ± 5.5	‐	16 ± 5	Mean: 508 days	The highest 30 s sample of 1 min intervals during exercise	Treadmill	Ventilation equivalent of oxygen, HR at rest, maximum SBP and ICD
Szabo 2011^69^	105 (105/0)	62 ± 1	24.7 ± 0.4	28 ± 2	18.2 ± 7.0	2 years	Modified Norton protocol	Treadmill	‐
Tseliou 2014^70^	80 (78/2)	57.8 ± 12.5	‐	57.8 ± 12.5	‐	6 months (mean: 286 ± 355 days)	Not reported	Not specified	‐
Van Iterson 2021^6^	329 (250/79)	63 ± 7	28.8 ± 5.7	23 ± 9	12.6 ± 3.7	1 year	Modified Naughton or Naughton protocol	Treadmill	Spirometry phenotype
Vecchiato 2024^71^	190 (158/32)	55.74 ± 13.04	26.99 ± 4.88	28.53 ± 6.45	15.02 ± 5.11	2.51 ± 1.17 years	The highest value of VO_2_ attained in a 30 s interval	Bicycle ergometer	Age, gender, LVEF, PET CO_2_max, no RER overshoot and VE/VCO_2_ slope
Walsh 1997^72^	84 (74/10)	62.7 ± 24.1	‐	‐	14 ± 12.9	710 days	Modified Bruce protocol	Treadmill	‐
Woods 2011^73^	132 (88/44)	56 ± 12	28.3 ± 4.7	29 ± 11	19.0 ± 5.9	62.4 months	Not reported	Treadmill	‐
Yan 2013^74^	224 (160/64)	68.8 ± 9.0	24.3 ± 3.2	53.7 ± 5.2	17.2 ± 4.6	30 months	Highest average values of a 10–15 s interval during exercise testing	Bicycle ergometer	Age, NYHA class, AF, DM, LVDd, BNP and VE/VCO_2_ slope
Zhuang 2021^75^	547 (447/100)	62.7 ± 9.7	‐	45 ± 11.9	14.64 ± 3.38	Median: 519 days	Not reported	Not specified	Age, AF, PCI, DM, HR at the eighth minute, CRP and uric acid

*Note*: Data are expressed as mean ± standard deviation.

Abbreviations: ACEI, angiotensin‐converting enzyme inhibitor; ACSM, The American College of Sports Medicine; AF, atrial fibrillation; ARB, angiotensin receptor blocker; ARNI, angiotensin receptor–neprilysin inhibitor; BMI, body mass index; BNP, B‐type natriuretic peptide; CAD, coronary artery disease; CO, cardiac output; COPD, chronic obstructive pulmonary disease; CRT, cardiac resynchronization therapy; DBP, diastolic blood pressure; DM, diabetes mellitus; EF, ejection fraction; eGFR, estimated glomerular filtration rate; Hb, haemoglobin; HDL, high‐density lipoprotein; HF, heart failure; hs‐CRP, high‐sensitivity C‐reactive protein; ICD, implantable cardioverter–defibrillator; IVCD, intraventricular conduction delay; LAD, left atrial diameter; LVDd, left ventricular diastolic dimension; LVDs, left ventricular systolic dimension; LVED, left ventricular end‐diastolic dimension; LVEF, left ventricular ejection fraction; LVESD, left ventricular end‐systolic dimension; LVMI, left ventricular mass index; MI, myocardial infarction; Na, sodium level; NT‐proBNP, N‐terminal pro B‐type natriuretic peptide; NYHA, New York Heart Association; O_2_ pulse, oxygen pulse; PCI, percutaneous coronary intervention; PET, pulmonary exercise testing; PVD, peripheral vascular disease; RER, respiratory exchange ratio; RHR, resting heart rate; RVEF, right ventricular ejection fraction; SBP, systolic blood pressure; VE/CO_2_, ventilation equivalent for carbon dioxide; VE/VCO_2_ slope, ventilatory efficiency/ventilatory response to carbon dioxide production slope.

### VO_2_peak and all‐cause mortality

**Figure 2:**
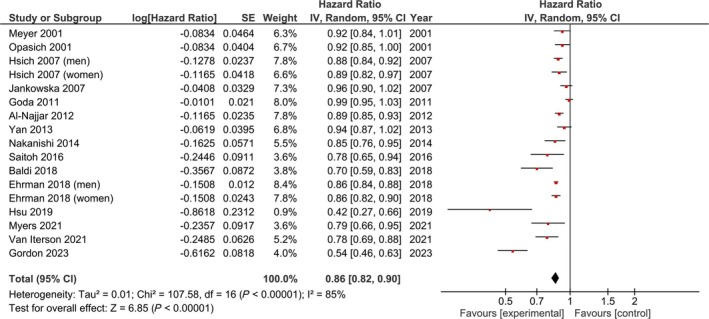
Adjusted impact of VO_2_peak on all‐cause mortality per 1 mL/kg/min increase in VO_2_peak.

Our main analysis showed that per 1 mL/kg/min increase in VO_2_peak, all‐cause mortality was significantly reduced (adjusted: HR: 0.86, 95% CI 0.82–0.90, *I* ^2^ = 85%, *P* < 0.01, *Figure* *2*; unadjusted: HR: 0.97, 95% CI 0.97–0.98, *I* ^2^ = 94%, *P* < 0.01, *Figure* S1).

Subgroup analysis based on age showed no differences between those 60 years and above (adjusted: HR: 0.86, 95% CI 0.81–0.92, *I* ^2^ = 73%, *P* < 0.01, *Figure* S2; unadjusted: HR: 0.97, 95% CI 0.96–0.98, *I* ^2^ = 95%, *P* < 0.01, *Figure* S3) and those below 60 years of age (adjusted: HR: 0.90, 95% CI 0.84–0.96, *I* ^2^ = 87%, *P* < 0.01, *Figure* S2; unadjusted: HR: 0.92, 95% CI 0.86–0.98, *I* ^2^ = 95%, *P* = 0.01, *Figure* S3). Identical findings were shown between those with a mean BMI < 25 kg/m^2^ (adjusted: HR: 0.90, 95% CI 0.82–0.99, *I* ^2^ = 52%, *P* = 0.04, *Figure* S4; unadjusted: HR: 0.89, 95% CI 0.84–0.94, *I* ^2^ = 64%, *P* < 0.01, *Figure* S5) and BMI > 25 kg/m^2^ (adjusted: HR: 0.88, 95% CI 0.83–0.93, *I* ^2^ = 86%, *P* < 0.01, *Figure* S4; unadjusted: HR: 0.97, 95% CI 0.96–0.98, *I* ^2^ = 96%, *P* < 0.01, *Figure* S5).

In addition, no changes were observed when we categorized our groups based on mean baseline VO_2_peak: <14 mL/kg/min (adjusted: HR: 0.74, 95% CI 0.59–0.95, *I* ^2^ = 92%, *P* = 0.02, *Figure* S6; unadjusted: HR: 0.89, 95% CI 0.83–0.96, *I* ^2^ = 95%, *P* < 0.01, *Figure* S7) and ≥14 mL/kg/min (adjusted: HR: 0.89, 95% CI 0.86–0.92, *I* ^2^ = 55%, *P* < 0.01, *Figure* S6; unadjusted: HR: 0.90, 95% CI 0.88–0.91, *I* ^2^ = 26%, *P* < 0.01, *Figure* S7).

**Figure 3:**
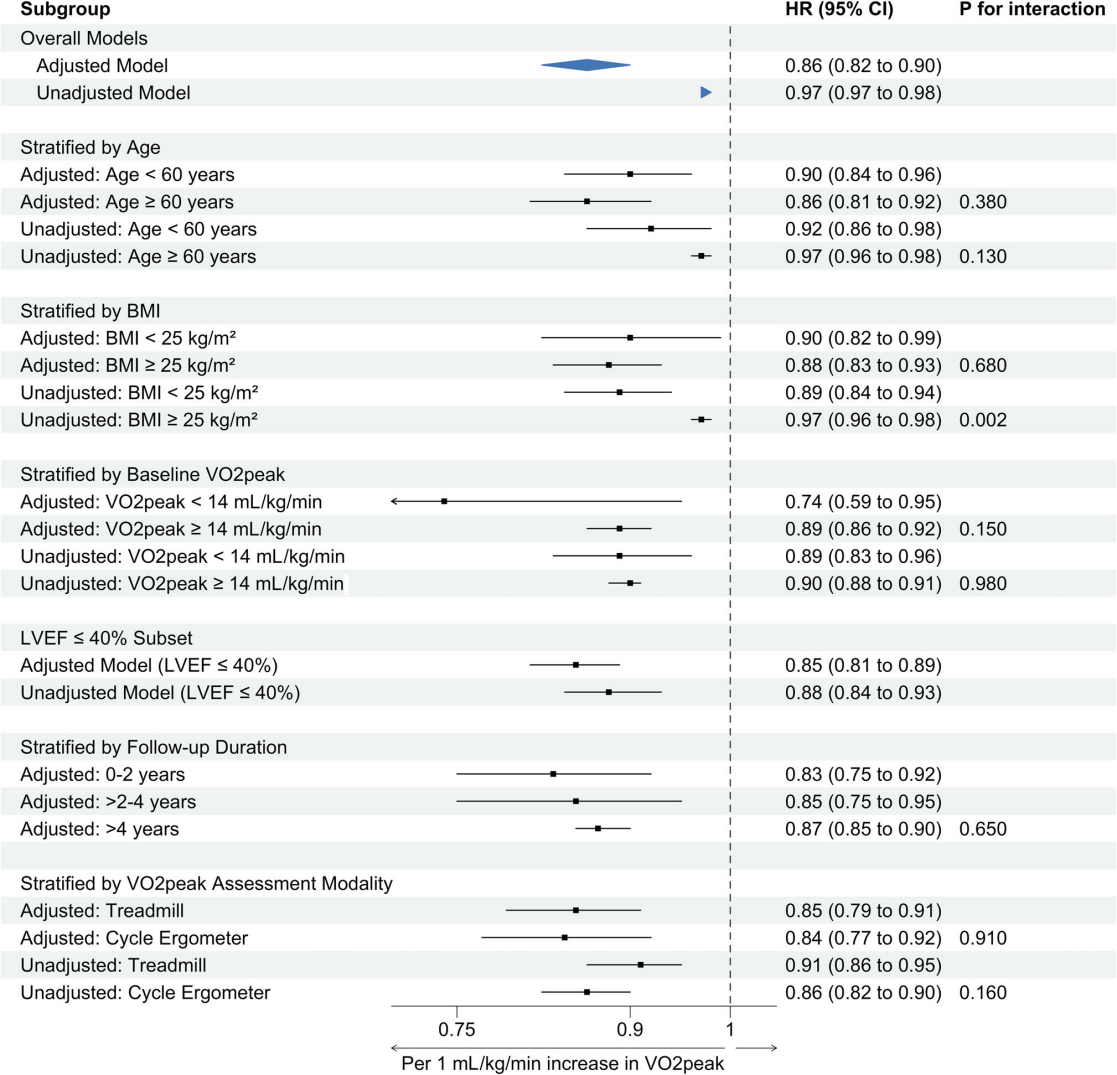
Subgroup analyses of the association between VO_2_peak and all‐cause mortality.

Similar findings were observed after accounting for participants with LVEF 40% and below (adjusted: HR: 0.85, 95% CI 0.81–0.89, *I* ^2^ = 86%, *P* < 0.01, *Figure* S8; unadjusted: HR: 0.88, 95% CI 0.84–0.93, *I* ^2^ = 95%, *P* < 0.01) (*Figure* S9). When we categorized our group based on different median follow‐up durations, no difference was observed (adjusted: 0–2 years: HR: 0.83, 95% CI 0.75–0.92, *I* ^2^ = 71%, *P* < 0.01; >2–4 years: HR: 0.85, 95% CI 0.75–0.95, *I* ^2^ = 92%, *P* < 0.01; and >4 years: HR: 0.87, 95% CI 0.85–0.90, *I* ^2^ = 59%, *P* < 0.01) (*Figure* S10). An overview of all subgroup analyses related to all‐cause mortality is presented in *Figure* *3*. In a separate analysis using a dichotomous threshold, individuals with VO_2_peak ≤14 mL/kg/min had a significantly higher risk of all‐cause mortality compared with those with higher VO_2_peak (HR: 2.55, 95% CI 1.77–3.67, *I* ^2^ = 0%, *P* < 0.01, *Figure* S11).

Excluding studies with a high risk of bias did not alter the findings of the main analysis using studies with adjusted models (HR: 0.86, 95% CI 0.82–0.90, *I* ^2^ = 87%, *P* < 0.01) (*Figure* S12).

### VO_2_peak and CV mortality

**Figure 4:**
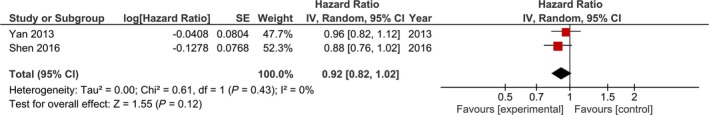
Adjusted impact of VO_2_peak on cardiovascular mortality per 1 mL/kg/min increase in VO_2_peak.

The main analysis using adjusted models showed no statistical significance of VO_2_peak with CV mortality (HR: 0.92, 95% CI 0.82–1.02, *I* ^2^ = 0%, *P* = 0.12) (*Figure* *4*) resulting from two studies. When the analysis was conducted using the unadjusted models from five studies, a significant prognostic link was observed (HR: 0.86, 95% CI 0.84–0.88, *I* ^2^ = 0%, *P* < 0.01) (*Figure* S13). VO_2_peak below 50% was significantly associated with an elevated hazard risk of CV mortality (*k* = 2; HR: 3.50, 95% CI 2.33–5.26, *I* ^2^ = 27%, *P* < 0.01) (*Figure* S14).

### VO_2_peak and VAD implantation, heart transplant and all‐cause mortality

**Figure 5:**
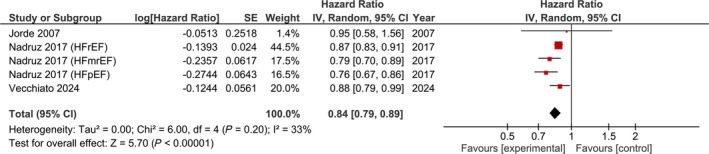
Adjusted impact of VO_2_peak on the risk of ventricular assist device implantation, heart transplant and all‐cause mortality per 1 mL/kg/min increase in VO_2_peak.

Higher VO_2_peak (per 1 mL/kg/min) was a significant contributor to reductions in incident VAD implantation, heart transplant and all‐cause mortality (adjusted: HR: 0.84, 95% CI 0.79–0.89, *I* ^2^ = 33%, *P* < 0.01, *Figure* *5*; unadjusted: HR: 0.84, 95% CI 0.81–0.86, *I* ^2^ = 7%, *P* < 0.01, *Figure* S15).

### Treadmill versus cycle ergometer: VO_2_peak and all‐cause mortality

In studies using treadmill, per 1 mL/kg/min increase in VO_2_peak, all‐cause mortality was significantly reduced (adjusted: HR: 0.85, 95% CI 0.79–0.91, *I* ^2^ = 92%, *P* < 0.01, *Figure* S16; unadjusted: HR: 0.91, 95% CI 0.86–0.95, *I* ^2^ = 96%, *P* < 0.01, *Figure* S17). Similar findings were shown when studies used a cycle ergometer (adjusted: HR: 0.84, 95% CI 0.77–0.92, *I* ^2^ = 75%, *P* < 0.01, *Figure* S16; unadjusted: HR: 0.86, 95% CI 0.82–0.90, *I* ^2^ = 57%, *P* < 0.01, *Figure* S17). The studies by Hsich *et al*. and Tseliou *et al*. did not mention which tool they used, while Piepoli *et al*. mentioned that either a treadmill or a cycle ergometer was employed. Therefore, they were not accounted for in the analysis.

### Other outcomes

The prognostic impact of VO_2_peak on all‐cause death or HF rehospitalization; VAD implantation, heart transplant, CV mortality and HF hospitalization; all‐cause mortality and HF hospitalization; CV mortality and HF rehospitalization; heart transplant and all‐cause mortality; heart transplant and CV mortality; and HF hospitalization is shown in *Table* S3.

### Publication bias

Egger's test showed a significant risk of publication bias regarding all‐cause mortality, indicating that the overall effect size may potentially overestimate or underestimate its true effect (*P* < 0.05) (*Table* S4 and *Figure* S18). Using the trim‐and‐fill method to calculate an adjusted effect size, we found a value of 0.8219 with 95% CI (0.7510–0.8928). The *Z* score for the effect size was 22.72 with *P* < 0.01, suggesting that VO_2_peak still has a statistically meaningful prognostic effect. Heterogeneity (*I* ^2^) among the studies was high, reaching 100% (*Table* S4 and *Figure* S19).

### Meta‐regression analysis

Variance among studies for the differences observed in multiple outcomes. Higher age was linked to increased all‐cause mortality (*r* = 0.017, 95% CI 0.01–0.02, *P* < 0.01), transplant and all‐cause mortality (*r* = 0.018, 95% CI 0.01–0.03, *P* < 0.01), and HF hospitalization (*r* = 0.017, 95% CI 0.01–0.03, *P* < 0.01). In addition, a higher proportion of females was linked to increased all‐cause mortality (*r* = 1.225, 95% CI 1.09–1.36, *P* < 0.01), all‐cause mortality and HF hospitalization (*r* = 1.948, 95% CI 1.59–2.31, *P* < 0.01), transplant and all‐cause mortality (*r* = 1.358, 95% CI 1.16–1.56, *P* < 0.01), and HF hospitalization (*r* = 1.519, 95% CI 1.12–1.92, *P* < 0.01). Higher BMI was associated with increased all‐cause mortality risk (*r* = 0.037, 95% CI 0.03–0.04, *P* < 0.01), all‐cause mortality and HF hospitalization (*r* = 0.039, 95% CI 0.02–0.04, *P* < 0.01), transplant and all‐cause mortality (*r* = 0.034, 95% CI 0.02–0.05, *P* < 0.01), but not HF hospitalization (*r* = 0.036, 95% CI −0.01 to 0.08, *P* = 0.10). Higher LVEF rates were linked to higher all‐cause mortality (*r* = 0.030, 95% CI 0.03–0.03, *P* < 0.01), all‐cause mortality and HF hospitalization (*r* = 0.028, 95% CI 0.02–0.03, *P* < 0.01), transplant and all‐cause mortality (*r* = 0.029, 95% CI 0.02–0.03, *P* < 0.01), and HF hospitalization (*r* = 0.027, 95% CI 0.02–0.03, *P* < 0.01). In terms of comorbidities, the presence of atrial fibrillation was associated with increased all‐cause mortality (*r* = 5.214, 95% CI 2.78–7.65, *P* < 0.01), all‐cause mortality and HF hospitalization (*r* = 7.976, 95% CI 6.81–9.14, *P* < 0.01), and HF hospitalization (*r* = 3.137, 95% CI 1.98–4.30, *P* < 0.01), and hypertension was associated with higher all‐cause mortality (*r* = 1.431, 95% CI 1.18–1.68, *P* < 0.01) and HF hospitalization (*r* = 1.99, 95% CI 1.48–2.50, *P* < 0.01). Chronic kidney disease was linked to increased risk for HF hospitalization (*r* = 4.852, 95% CI 4.32–5.39, *P* < 0.01), while diabetes was linked to increased all‐cause mortality (*r* = 3.066, 95% CI 2.71–3.42, *P* < 0.01), transplant and all‐cause mortality (*r* = 3.745, 95% CI 3.15–4.34, *P* < 0.01), and HF hospitalization (*r* = 4.127, 95% CI 3.41–4.84, *P* < 0.01). Regarding medications, angiotensin‐converting enzyme inhibitors (ACEIs)/angiotensin receptor blockers (ARBs), beta‐blockers, diuretics and statins increased the risk for all‐cause mortality (ACEIs/ARBs: *r* = 0.015, 95% CI 0.01–0.02, *P* < 0.01; beta‐blockers: *r* = 0.016, 95% CI 0.01–0.02, *P* < 0.01; diuretics: *r* = 0.015, 95% CI 0.01–0.02, *P* < 0.01; and statins: *r* = 0.023, 95% CI 0.02–0.03, *P* < 0.01). A summary of meta‐regressions is presented in *Table* S5.

### Risk of bias

Fourteen studies were evaluated as having a high risk of bias,^17^, ^22^, ^27^, ^39^, ^40^, ^41^, ^43^, ^44^, ^64^, ^73^, ^78^, ^85^, ^86^, ^89^ while 37 studies had a moderate risk of bias. Scores ranged from 2 to 9, with common strengths among studies being the inclusion of adequate representation and selection of the intervention and exposed cohorts, respectively, and presentation of outcome of interest assessments. However, several studies lacked control for important confounding factors and had limited follow‐up adequacy, although their follow‐up was long enough for outcomes to occur. Details are presented in *Table* S6.

## Discussion

In this study, our principal finding was that increased VO_2_peak, primarily as a unit of change per 1 mL/kg/min, was linked to reduced all‐cause mortality, all‐cause mortality or HF rehospitalization, VAD implantation, heart transplant, CV mortality and HF hospitalization, CV mortality and HF rehospitalization, heart transplant and all‐cause mortality, and HF hospitalization. Our subgroups based on mean age (≥60 vs. <60 years), mean BMI (<25 vs. >25 kg/m^2^), mean baseline VO_2_peak (<14 vs. ≥14 mL/kg/min) and LVEF ≤ 40% did not alter the findings of the main analyses. Second, VO_2_peak below 50% significantly elevated the prognostic risk of CV mortality. Age, sex, BMI, LVEF and reported comorbidities were shown as mediators of this relationship.

Evidence for the secondary and composite outcomes was inconclusive. Associations between VO_2_ peak and (i) all‐cause mortality and HF hospitalization, (ii) heart transplant and CV mortality, and (iii) CV mortality alone did not reach statistical significance. Each analysis drew on no more than three studies, which produced wide CIs and moderate to high heterogeneity, which are features indicative of limited statistical power. It is worth noting that the unadjusted analysis in relation to these outcomes displayed a significant result. Therefore, although our adjusted models accounted for key covariates, the influence of unmeasured or imprecisely measured confounders should not be excluded.

The aforementioned findings were based on the values provided through adjusted statistical models acquired from the included studies. Our findings reinforce the role of VO_2_peak as an independent prognostic marker for HF patients. As a non‐invasive but imperfect surrogate of peak cardiac output,^47^, ^48^ VO_2_peak is pivotal in assessing patients for heart transplantation and VAD implantation, as well as in evaluating the impact of pharmacological interventions.^78^ Although the predictive power of VO_2_peak has its limitations, its utility might be further enhanced by incorporating additional factors, such as CPET variables.^50^, ^93^ However, our analysis, conducted on models adjusted for a variety of confounding factors, supports the established value of VO_2_peak as a reliable predictor of both short‐term and long‐term mortality.

Although our meta‐analysis shows that each 1 mL/kg/min increment in VO_2_peak is associated with a 14% lower adjusted mortality, reliance on a single maximal‐effort value is vulnerable to submaximal effort and protocol variation. To improve prognostic precision, recent algorithms pair CPET indices with resting variables. The MECKI score, for example, combines per cent‐predicted VO_2_peak and the VE/VCO_2_ slope with haemoglobin, serum sodium, MDRD–estimated glomerular filtration rate and left ventricular ejection fraction, yielding *C* statistics of ~0.80 at 1 year in both derivation and validation cohorts.^80^ Embedding our continuous slope within such multiparametric tools enables two clinically relevant applications: first, refining timing of referral for transplant or VAD when VO_2_peak declines to ≤14 mL/kg/min (≤12 mL/kg/min in patients on beta‐blocker) or ≤50% predicted, thresholds endorsed by the international guidelines.^49^, ^53^ Second, expressing VO_2_peak as a continuous variable allows clinicians to determine whether observed improvements or declines reflect meaningful shifts in risk, supporting dynamic risk stratification beyond fixed threshold‐based decisions.

Mechanistically, increased VO_2_peak reflects greater CV function that is associated with improved skeletal muscle metabolism and overall physical capacity.^54^, ^55^, ^57^ Considering that patients with HF are characterized by accelerating muscle wasting accompanied by higher concentrations of oxidative stress and impaired mitochondrial function, increased VO_2_peak may reflect greater mitochondrial structure, oxidative capacity and substrate utilization (e.g., improved insulin sensitivity).^58^, ^59^, ^60^, ^62^ These factors may augment physical capacity and subsequent engagement with physical activity, key features of higher functionality and quality of life. Moreover, HF patients with higher VO_2_peak levels may experience fewer CV events, including myocardial infarction, arrhythmias and stroke, encompassing decreased rates of HF hospitalizations.^89^ Higher VO_2_peak has been linked to improved clinical outcomes after cardiac transplantation, highlighting its potential as an indicator of therapeutic efficacy or preventative measure.^90^

Cardiac rehabilitation programmes, including structured exercise and pharmacological therapies targeting neurohormonal pathways, have shown promising results in augmenting VO_2_peak and subsequent clinical outcomes, improving quality of life.^7^, ^66^ Importantly, individualized exercise prescriptions tailored to patients' health and functional status and comorbidities are paramount to optimizing their management. Aerobic exercise may improve cardiac contractility and reduce oxygen demand, enhancing cardiac output and endothelial and peripheral vascular function.^92^ In addition, exercise‐induced vasodilation and angiogenesis improve blood flow distribution, alleviating myocardial ischaemia and reducing the risk of adverse CV events.^93^ Exercise may also lower circulating catecholamine and proinflammatory cytokine concentrations, ameliorating fibrosis, which is associated with HF progression.^94^ Finally, HF hospitalizations may be reduced due to the effects of (primarily aerobic) exercise in improving patients' lipid profile,^95^ which may mitigate atherosclerosis progression and the number of CV events.^96^

Our meta‐regression analyses corroborate with the clinical significance of existing comorbidities and patients' age, BMI and LVEF status that may all mediate (in)directly the relationship between VO_2_peak and prognostic clinical outcomes. The impact of these parameters leading to increased mortality and hospitalizations may exceed the metabolic benefits induced by higher VO_2_peak, considering the various degrees of individual capacity a patient may be engaged in cardiac rehabilitation programmes. Therefore, a holistic approach taking these factors into account during HF management should be considered.

### Limitations

Our study has several limitations. Although we ran a separate analysis for all‐cause mortality on LVEF ≤ 40%, our findings are not applicable to a specific HF phenotype, limiting the external validity of the conclusions across HF populations. Furthermore, we do not have sufficient data to provide reliable results based on specific follow‐up durations, which were only analysed for all‐cause mortality. In addition, studies adjusted for different covariates, which may hamper the accuracy of the meta‐analyses, while simultaneously, discrepancies were observed with regard to different VO_2_peak protocols among studies. Importantly, our analysis was constrained by the lack of individual patient data, which limited our ability to perform more detailed subgroup analyses and account for individual‐level variations. For instance, subgroup analyses based on different age and BMI ranges, as well as sex, could have provided further insights on the prognostic impact of VO_2_peak on clinical outcomes, particularly if that was combined with known HF phenotypes. Additionally, while we focused on VO_2_peak as a continuous variable, routinely used clinical cut‐offs such as the Weber classification and <50% predicted VO_2_peak, which account for age and gender, were sparingly employed in the included research studies. Nonetheless, we ensured that every study utilizing these categorical measures was included in our analysis.

## Conclusions

This study highlights VO_2_peak as a prognostic marker for multiple causes of mortality and hospitalization in patients with HF. Through multiple meta‐analyses, we affirm that VO_2_peak serves as a predictor of adverse clinical outcomes, offering clinicians insights into patient risk stratification and facilitating targeted management. The findings highlight the potential of incorporating VO_2_peak assessment into routine clinical practice, which may help with tailored therapeutic interventions in this patient group. Future studies should explicitly explore the prognostic impact of VO_2_peak on different HF phenotypes.

## Conflict of interest statement

GYHL is a consultant and speaker for BMS/Pfizer, Boehringer Ingelheim, Daiichi Sankyo and Anthos. No fees are received personally. He is a National Institute for Health and Care Research (NIHR) Senior Investigator and co‐PI of the AFFIRMO project on multimorbidity in AF (Grant Agreement No. 899871), TARGET project on digital twins for personalized management of atrial fibrillation and stroke (Grant Agreement No. 101136244) and ARISTOTELES project on artificial intelligence for management of chronic long‐term conditions (Grant Agreement No. 101080189), which are all funded by Horizon Europe's Research and Innovation Programme. The other authors declare no conflicts of interest.

## Supporting information


**Data S1.** Supporting information.

**Table S1.** Key terms employed in the screening of the literature search.

**Table S2.** Studies included in the meta‐analysis by outcome.

**Table S3.** Prognostic impact of VO_2_peak on using adjusted models.

**Table S4.** Egger's test and Trim and Fill method.

**Table S5.** Meta‐regression analyses based on age, sex, BMI, LVEF, and reported comorbidities.

**Table S6.** Risk of bias and quality assessment of the included studies.

**Figure S1.** Unadjusted impact of VO_2_peak on all‐cause mortality per 1 ml/kg/min increase in VO_2_peak.

**Figure S2.** Adjusted impact of VO_2_peak on all‐cause mortality per 1 ml/kg/min increase in VO_2_peak, stratified by age.

**Figure S3.** Unadjusted impact of VO_2_peak on all‐cause mortality per 1 ml/kg/min increase in VO_2_peak, stratified by age.

**Figure S4.** Adjusted impact of VO_2_peak on all‐cause mortality per 1 ml/kg/min increase in VO_2_peak, stratified by BMI.

**Figure S5.** Unadjusted impact of VO_2_peak on all‐cause mortality per 1 ml/kg/min increase in VO_2_peak, stratified by BMI.

**Figure S6.** Adjusted impact of VO_2_peak on all‐cause mortality per 1 ml/kg/min increase in VO_2_peak, stratified by baseline VO_2_peak levels.

**Figure S7.** Unadjusted impact of VO_2_peak on all‐cause mortality per 1 ml/kg/min increase in VO_2_peak, stratified by baseline VO_2_peak levels.

**Figure S8.** Adjusted impact of VO_2_peak on all‐cause mortality per 1 ml/kg/min increase in VO_2_peak among participants with LVEF ≤ 40%.

**Figure S9.** Unadjusted impact of VO_2_peak on all‐cause mortality per 1 ml/kg/min increase in VO_2_peak among participants with LVEF ≤ 40%.

**Figure S10.** Adjusted impact of VO_2_peak on all‐cause mortality per 1 ml/kg/min increase in VO_2_peak, categorized by different follow‐up durations.

**Figure S11.** Risk of all‐cause mortality associated with VO_2_peak equal or below 14 ml/kg/min.

**Figure S12.** Impact of VO_2_peak on all‐cause mortality per 1 ml/kg/min increase in VO_2_peak, with exclusion of high‐risk of bias studies.

**Figure S13.** Unadjusted impact of VO_2_peak on cardiovascular mortality per 1 ml/kg/min increase in VO_2_peak.

**Figure S14.** Risk of cardiovascular mortality associated with VO_2_peak below 50% of predicted value.

**Figure S15.** Unadjusted impact of VO_2_peak on incident ventricular assist device implantation, heart transplant, and all‐cause mortality per 1 ml/kg/min increase in VO_2_peak.

**Figure S16.** Adjusted impact of VO_2_peak on all‐cause mortality based on studies using treadmill vs. cycle ergometer.

**Figure S17.** Unadjusted impact of VO_2_peak on all‐cause mortality based on studies using treadmill vs. cycle ergometer.

**Figure S18.** Funnel plot of the included studies.

**Figure S19.** Assessment of publication bias using the Trim and Fill method.

## Data Availability

Data are available upon reasonable request.
