# Supplementary material for: Prognostic impact of peak oxygen consumption in heart failure: A systematic review and meta‐analysis
Source: ESC Heart Fail. 2025 Aug 12;12(5):3624–42. doi: 10.1002/ehf2.15391 (PMC12450781; doi:10.1002/ehf2.15391)
Supplement: Supplementary file 2 — Table S1. Key terms employed in the screening of the literature search. [file EHF2-12-3624-s011.docx]

**Table S1.**Key terms employed in the screening of the literature search.

| **Database** | **Search terms** |
| --- | --- |
|  |  |
| PubMed | (“vo2max” OR “vo2 peak” OR “vo2peak” OR “vo2 max” OR Aerobic OR Endurance OR Maximal Oxygen Uptake OR  maximal oxygen consumption OR maximal oxygen capacity) AND (mortality OR prognos* OR death OR survival OR hazard*)  AND (“heart failure” OR “heart transplant*” OR “cardiac transplant*”) |
| Cochrane Library | (“vo2max” OR “vo2 peak” OR “vo2peak” OR “vo2 max” OR Aerobic OR Endurance OR Maximal Oxygen Uptake OR  maximal oxygen consumption OR maximal oxygen capacity) AND (mortality OR prognos* OR death OR survival OR hazard*)  AND (“heart failure” OR “heart transplant*” OR “cardiac transplant*”) |
| Web of Science | (“vo2max” OR “vo2 peak” OR “vo2peak” OR “vo2 max” OR Aerobic OR Endurance OR Maximal Oxygen Uptake OR  maximal oxygen consumption OR maximal oxygen capacity) AND (mortality OR prognos* OR death OR survival OR hazard*)  AND (“heart failure” OR “heart transplant*” OR “cardiac transplant*”) |
| Scopus | (“vo2max” OR “vo2 peak” OR “vo2peak” OR “vo2 max” OR Aerobic OR Endurance OR Maximal Oxygen Uptake OR  maximal oxygen consumption OR maximal oxygen capacity) AND (mortality OR prognos* OR death OR survival OR hazard*)  AND (“heart failure” OR “heart transplant*” OR “cardiac transplant*”) |
