# Supplementary material for: Prognostic impact of peak oxygen consumption in heart failure: A systematic review and meta‐analysis
Source: ESC Heart Fail. 2025 Aug 12;12(5):3624–42. doi: 10.1002/ehf2.15391 (PMC12450781; doi:10.1002/ehf2.15391)
Supplement: Supplementary file 3 — Table S2. Studies included in the meta‐analysis by outcome. [file EHF2-12-3624-s015.docx]

**Table S2.** Studies included in the meta-analysis by outcome.

| **Outcome** | **Number of Studies** | **References** |
| --- | --- | --- |
| All-cause mortality | 28 | (1-28) |
| CV mortality | 6 | (8, 29-33) |
| VAD, transplant, and all-cause mortality | 5 | (34-38) |
| All-cause mortality and HF rehospitalization | 2 | (39, 40) |
| VAD, transplant, CV mortality, and HF hospitalization | 3 | (1, 41, 42) |
| All-cause mortality and HF hospitalization | 6 | (23, 43-47) |
| CV mortality and HF rehospitalization | 5 | (3, 48-51) |
| Transplant and all-cause mortality | 8 | (52-59) |
| Transplant and CV mortality | 3 | (60-62) |
| HF hospitalization | 3 | (37, 63, 64) |

1. Myers J, de Souza e Silva CG, Arena R, Kaminsky L, Christle JW, Busque V, et al. Comparison of the FRIEND and Wasserman‐Hansen Equations in Predicting Outcomes in Heart Failure. Journal of the American Heart Association. 2021;10(21):e021246.

2. Al-Najjar Y, Witte KK, Clark AL. Chronotropic incompetence and survival in chronic heart failure. International journal of cardiology. 2012;157(1):48-52.

3. Baldi C, Citro R, Silverio A, Di Maio M, De Rosa R, Bonadies D, et al. Predictors of outcome in heart failure patients with severe functional mitral regurgitation undergoing MitraClip treatment. International Journal of Cardiology. 2019;284:50-8.

4. Gordon J, Michelis KC, Pandey A, Ayers C, Thibodeau JT, Grodin JL, et al. Oxygen uptake efficiency slope and prognosis in heart failure with reduced ejection fraction. The American Journal of Cardiology. 2023;201:273-80.

5. Goda A, Williams P, Mancini D, Lund LH. Selecting patients for heart transplantation: comparison of the Heart Failure Survival Score (HFSS) and the Seattle heart failure model (SHFM). The Journal of heart and lung transplantation. 2011;30(11):1236-43.

6. Hsich E, Chadalavada S, Krishnaswamy G, Starling RC, Pothier CE, Blackstone EH, et al. Long-term prognostic value of peak oxygen consumption in women versus men with heart failure and severely impaired left ventricular systolic function. The American journal of cardiology. 2007;100(2):291-5.

7. Meyer FJ, Borst MM, Zugck C, Kirschke A, Schellberg D, Kübler W, et al. Respiratory muscle dysfunction in congestive heart failure: clinical correlation and prognostic significance. Circulation. 2001;103(17):2153-8.

8. Yan J, Gong S-J, Li L, Yu H-Y, Dai H-W, Chen J, et al. Combination of B-type natriuretic peptide and minute ventilation/carbon dioxide production slope improves risk stratification in patients with diastolic heart failure. International journal of cardiology. 2013;162(3):193-8.

9. Hsu C-C, Fu T-C, Yuan S-S, Wang C-H, Liu M-H, Shyu Y-C, et al. High-intensity interval training is associated with improved long-term survival in heart failure patients. Journal of clinical medicine. 2019;8(3):409.

10. Gitt AK, Wasserman K, Kilkowski C, Kleemann T, Kilkowski A, Bangert M, et al. Exercise anaerobic threshold and ventilatory efficiency identify heart failure patients for high risk of early death. Circulation. 2002;106(24):3079-84.

11. Hansen A, Haass M, Zugck C, Krueger C, Unnebrink K, Zimmermann R, et al. Prognostic value of Doppler echocardiographic mitral inflow patterns: implications for risk stratification in patients with chronic congestive heart failure. Journal of the American College of Cardiology. 2001;37(4):1049-55.

12. Opasich C, Pinna G, Mazza A, Febo O, Riccardi R, Riccardi P, et al. Six-minute walking performance in patients with moderate-to-severe heart failure; is it a useful indicator in clinical practice? European heart journal. 2001;22(6):488-96.

13. Van Iterson EH, Cho L, Tonelli A, Finet JE, Laffin LJ. All‐cause mortality predicted by peak oxygen uptake differs depending on spirometry pattern in patients with heart failure and reduced ejection fraction. ESC Heart Failure. 2021;8(4):2731-40.

14. Jankowska EA, Witkowski T, Ponikowska B, Reczuch K, Borodulin‐Nadzieja L, Anker SD, et al. Excessive ventilation during early phase of exercise: A new predictor of poor long‐term outcome in patients with chronic heart failure. European Journal of Heart Failure. 2007;9(10):1024-31.

15. Nakanishi M, Takaki H, Kumasaka R, Arakawa T, Noguchi T, Sugimachi M, et al. Targeting of high peak respiratory exchange ratio is safe and enhances the prognostic power of peak oxygen uptake for heart failure patients. Circulation Journal. 2014;78(9):2268-75.

16. Saitoh M, Dos Santos MR, Ebner N, Emami A, Konishi M, Ishida J, et al. Nutritional status and its effects on muscle wasting in patients with chronic heart failure: insights from Studies Investigating Co-morbidities Aggravating Heart Failure. Wiener klinische Wochenschrift. 2016;128:497-504.

17. Ehrman JK, Brawner CA, Shafiq A, Lanfear DE, Saval M, Keteyian SJ. Cardiopulmonary exercise measures of men and women with HFrEF differ in their relationship to prognosis: the Henry ford hospital cardiopulmonary exercise testing (FIT-CPX) project. Journal of cardiac failure. 2018;24(4):227-33.

18. Czubaszewski Ł, Straburzyńska-Lupa A, Migaj J, Straburzyńska-Migaj E. Comparison of prognostic values of cardiopulmonary and heart rate parameters in exercise testing in men with heart failure. Cardiology Journal. 2018;25(6):701-8.

19. Doehner W, Rauchhaus M, Ponikowski P, Godsland IF, von Haehling S, Okonko DO, et al. Impaired insulin sensitivity as an independent risk factor for mortality in patients with stable chronic heart failure. Journal of the American College of Cardiology. 2005;46(6):1019-26.

20. Ingle L, Sloan R, Carroll S, Goode K, Cleland JG, Clark AL. Influence of body mass on risk prediction during cardiopulmonary exercise testing in patients with chronic heart failure. Experimental & Clinical Cardiology. 2012;17(4):179.

21. Koike A, Koyama Y, Itoh H, Adachi H, Marumo F, Hiroe M. Prognostic significance of cardiopulmonary exercise testing for 10-year survival in patients with mild to moderate heart failure. Japanese circulation journal. 2000;64(12):915-20.

22. Piepoli MF, Corrà U, Veglia F, Bonomi A, Salvioni E, Cattadori G, et al. Exercise tolerance can explain the obesity paradox in patients with systolic heart failure: data from the MECKI Score Research Group. European journal of heart failure. 2016;18(5):545-53.

23. Scardovi AB, De Maria R, Ferraironi A, Gatto L, Celestini A, Forte S, et al. A case for assessment of oscillatory breathing during cardiopulmonary exercise test in risk stratification of elderly patients with chronic heart failure. International journal of cardiology. 2012;155(1):115-9.

24. Szabo T, von Haehling S, Habedank D, Rauchhaus M, Lainscak M, Sandek A, et al. Usefulness of minimal modelling to assess impaired insulin sensitivity in patients with chronic heart failure. International journal of cardiology. 2011;147(1):47-51.

25. Walsh JT, Charlesworth A, Andrews R, Hawkins M, Cowley AJ. Relation of daily activity levels in patients with chronic heart failure to long-term prognosis. The American journal of cardiology. 1997;79(10):1364-9.

26. Tseliou E, Terrovitis JV, Kaldara EE, Ntalianis AS, Repasos E, Katsaros L, et al. Red blood cell distribution width is a significant prognostic marker in advanced heart failure, independent of hemoglobin levels. Hellenic J Cardiol. 2014;55(6):457-61.

27. Chen S-M, Wu M-K, Chen C, Wang L-Y, Guo N-W, Wei C-L, et al. Benefit of cardiac rehabilitation in acute heart failure patients with cognitive impairment. Heliyon. 2024;10(9).

28. Li JP, Slocum C, Sbarbaro J, Schoenike M, Campain J, Prasad C, et al. Percent predicted peak exercise oxygen pulse provides insights into ventricular-vascular response and prognosticates HFpEF. JACC: Advances. 2024;3(8):101101.

29. Magri D, Piepoli M, Corra U, Gallo G, Maruotti A, Vignati C, et al. Cardiovascular death risk in recovered mid-range ejection fraction heart failure: insights from cardiopulmonary exercise test. Journal of Cardiac Failure. 2020;26(11):932-43.

30. Malhotra R, Dhakal BP, Eisman AS, Pappagianopoulos PP, Dress A, Weiner RB, et al. Pulmonary vascular distensibility predicts pulmonary hypertension severity, exercise capacity, and survival in heart failure. Circulation: Heart Failure. 2016;9(6):e003011.

31. Shen Y, Song H, Ma W, Gong Z, Ni Y, Zhang X, et al. The prognostic value of peak cardiac power output in Chinese patients with chronic heart failure. Plos one. 2016;11(1):e0147423.

32. de Groote P, Dagorn J, Soudan B, Lamblin N, McFadden E, Bauters C. B-type natriuretic peptide and peak exercise oxygen consumption provide independent information for risk stratification in patients with stable congestive heart failure. Journal of the American College of Cardiology. 2004;43(9):1584-9.

33. Lamblin N, Mouquet F, Hennache B, Dagorn J, Susen S, Bauters C, et al. High-sensitivity C-reactive protein: potential adjunct for risk stratification in patients with stable congestive heart failure. European Heart Journal. 2005;26(21):2245-50.

34. Guazzi M, Myers J, Peberdy MA, Bensimhon D, Chase P, Arena R. Maximal dyspnea on exertion during cardiopulmonary exercise testing is related to poor prognosis and echocardiography with tissue Doppler imaging in heart failure. Congestive Heart Failure. 2009;15(6):277-83.

35. Jorde UP, Colombo PC, Ahuja K, Hudaihed A, Onat D, Diaz T, et al. Exercise-induced increases in oxidized low-density lipoprotein are associated with adverse outcomes in chronic heart failure. Journal of cardiac failure. 2007;13(9):759-64.

36. Lala A, Shah KB, Lanfear DE, Thibodeau JT, Palardy M, Ambardekar AV, et al. Predictive value of cardiopulmonary exercise testing parameters in ambulatory advanced heart failure. Heart Failure. 2021;9(3):226-36.

37. Nadruz Jr W, West E, Sengeløv M, Santos M, Groarke JD, Forman DE, et al. Prognostic value of cardiopulmonary exercise testing in heart failure with reduced, midrange, and preserved ejection fraction. Journal of the American Heart Association. 2017;6(11):e006000.

38. Vecchiato M, Neunhaeuserer D, Zanardo E, Quinto G, Battista F, Aghi A, et al. Respiratory exchange ratio overshoot during exercise recovery: a promising prognostic marker in HFrEF. Clinical Research in Cardiology. 2024:1-12.

39. Lee M-F, Chen W-S, Fu T-C, Liu M-H, Wang J-S, Hsu C-C, et al. Non-invasive cardiac index monitoring during cardiopulmonary functional testing provides additional prognostic value in patients after acute heart failure. International Heart Journal. 2012;53(6):364-9.

40. Zhuang B, Shen T, Li D, Jiang Y, Li G, Luo Q, et al. A model for the prediction of mortality and hospitalization in Chinese heart failure patients. Frontiers in Cardiovascular Medicine. 2021;8:761605.

41. Cunha GJ, Maltês S, Rocha BM, Nina D, Aguiar C, Andrade MJ, et al. Beyond exercise oscillatory ventilations: the prognostic impact of loop gain in heart failure. European Journal of Preventive Cardiology. 2023;30(6):498-505.

42. Chiang W-J, Lee J-T, Hung S-Y, Hsu P-C, Chou C-L. Prognostic Value of Body Surface Area-Adjusted Oxygen Uptake Efficiency Slope in Heart Failure Patients. Acta Cardiologica Sinica. 2024;40(3):322.

43. Badr Eslam R, Öztürk B, Rettl R, Capelle CDJ, Qin H, Binder C, et al. Impact of tafamidis and optimal background treatment on physical performance in patients with transthyretin amyloid cardiomyopathy. Circulation: Heart Failure. 2022;15(7):e008381.

44. Koerber DM, Rosenbaum AN, Olson TP, Kushwaha S, Stulak J, Maltais S, et al. Exercise-induced hypoxemia predicts heart failure hospitalization and death in patients supported with left ventricular assist devices. The International Journal of Artificial Organs. 2020;43(3):165-72.

45. Nakanishi M, Miura H, Irie Y, Nakao K, Fujino M, Otsuka F, et al. Association of adherence to a 3 month cardiac rehabilitation with long‐term clinical outcomes in heart failure patients. ESC Heart Failure. 2022;9(2):1424-35.

46. Silverii MV, Argirò A, Baldasseroni S, Fumagalli C, Zampieri M, Guerrieri L, et al. Prognostic value of cardiopulmonary exercise testing in patients with transthyretin cardiac amyloidosis. Internal and Emergency Medicine. 2023;18(2):585-93.

47. Murata M, Adachi H, Nakade T, Miyaishi Y, Kan H, Okonogi S, et al. Ventilatory Efficacy After Transcatheter Aortic Valve Replacement Predicts Mortality and Heart Failure Events in Elderly Patients. Circulation Journal. 2019;83(10):2034-43.

48. Sato T, Yamauchi H, Kanno Y, Suzuki S, Yoshihisa A, Yamaki T, et al. Comparisons of prognostic factors between young and elderly patients with chronic heart failure. Geriatrics & gerontology international. 2015;15(4):435-42.

49. Shibata A, Hanatani A, Izumi Y, Kitada R, Iwata S, Yoshiyama M. Serum brain-derived neurotrophic factor level and exercise tolerance complement each other in predicting the prognosis of patients with heart failure. Heart and vessels. 2018;33:1325-33.

50. Rossi A, Cicoira M, Bonapace S, Golia G, Zanolla L, Franceschini L, et al. Left atrial volume provides independent and incremental information compared with exercise tolerance parameters in patients with heart failure and left ventricular systolic dysfunction. Heart. 2007;93(11):1420-5.

51. Pugliese NR, Paneni F, Mazzola M, De Biase N, Del Punta L, Gargani L, et al. Impact of epicardial adipose tissue on cardiovascular haemodynamics, metabolic profile, and prognosis in heart failure. European Journal of Heart Failure. 2021;23(11):1858-71.

52. Shafiq A, Brawner CA, Aldred HA, Lewis B, Williams CT, Tita C, et al. Prognostic value of cardiopulmonary exercise testing in heart failure with preserved ejection fraction. The Henry Ford HospITal CardioPulmonary EXercise Testing (FIT-CPX) project. American heart journal. 2016;174:167-72.

53. Chen SM, Wu PJ, Wang LY, Wei CL, Cheng CI, Fang HY, et al. Optimizing exercise testing‐based risk stratification to predict poor prognosis after acute heart failure. ESC Heart Failure. 2023;10(2):895-906.

54. O’Neill JO, Young JB, Pothier CE, Lauer MS. Peak oxygen consumption as a predictor of death in patients with heart failure receiving β-blockers. Circulation. 2005;111(18):2313-8.

55. Kallistratos MS, Dritsas A, Laoutaris ID, Cokkinos DV. Incremental value of N-terminal pro–brain natriuretic peptide over left ventricle ejection fraction and aerobic capacity for estimating prognosis in heart failure patients. The Journal of heart and lung transplantation. 2008;27(11):1251-6.

56. Woods PR, Bailey KR, Wood CM, Johnson BD. Submaximal exercise gas exchange is an important prognostic tool to predict adverse outcomes in heart failure. European Journal of Heart Failure. 2011;13(3):303-10.

57. Myers J, Arena R, Oliveira RB, Bensimhon D, Hsu L, Chase P, et al. The lowest VE/VCO2 ratio during exercise as a predictor of outcomes in patients with heart failure. Journal of cardiac failure. 2009;15(9):756-62.

58. Romuk E, Jacheć W, Zbrojkiewicz E, Mroczek A, Niedziela J, Gąsior M, et al. Ceruloplasmin, NT-proBNP, and Clinical Data as Risk Factors of Death or Heart Transplantation in a 1-Year Follow-Up of Heart Failure Patients. Journal of Clinical Medicine. 2020;9(1):137.

59. Stolker JM, Heere B, Geltman EM, Schechtman KB, Peterson LR. Prospective comparison of ventilatory equivalent versus peak oxygen consumption in predicting outcomes of patients with heart failure. The American journal of cardiology. 2006;97(11):1607-10.

60. Brás PG, Gonçalves AV, Reis JF, Moreira RI, Pereira-da-Silva T, Rio P, et al. Cardiopulmonary Exercise Testing in the Age of New Heart Failure Therapies: Still a Powerful Tool? Biomedicines. 2023;11(8).

61. Corrà U, Giordano A, Piepoli M. Cardiopulmonary exercise testing in chronic heart failure patients treated with beta-blockers: still a valid prognostic tool. International Journal of Cardiology. 2020;317:128-32.

62. Hoyer D, Maestri R, TERESA LA ROVERE M, DOMENICO PINNA G. Autonomic response to cardiac dysfunction in chronic heart failure: a risk predictor based on autonomic information flow. Pacing and clinical electrophysiology. 2008;31(2):214-20.

63. Magrì D, Banfi C, Maruotti A, Farina S, Vignati C, Salvioni E, et al. Plasma immature form of surfactant protein type B correlates with prognosis in patients with chronic heart failure. A pilot single-center prospective study. International Journal of Cardiology. 2015;201:394-9.

64. Pugliese NR, Fabiani I, Mandoli GE, Guarini G, Galeotti GG, Miccoli M, et al. Echo-derived peak cardiac power output-to-left ventricular mass with cardiopulmonary exercise testing predicts outcome in patients with heart failure and depressed systolic function. European Heart Journal-Cardiovascular Imaging. 2019;20(6):700-8.
