# Supplementary material for: Prognostic impact of peak oxygen consumption in heart failure: A systematic review and meta‐analysis
Source: ESC Heart Fail. 2025 Aug 12;12(5):3624–42. doi: 10.1002/ehf2.15391 (PMC12450781; doi:10.1002/ehf2.15391)
Supplement: Supplementary file 4 — Table S3. Prognostic impact of VO2peak on using adjusted models. [file EHF2-12-3624-s014.docx]

**Table S3.** Prognostic impact of VO_2_peak on using adjusted and unadjusted models for different outcomes of interest.

| **Adjusted** | | | | | |
| --- | --- | --- | --- | --- | --- |
| Outcome | N of studies | HR | 95% CI | I^2^ | P value |
| All-cause mortality or HF  rehospitalization | 2 | 0.95 | 0.91 – 0.99 | 0% | 0.02* |
| VAD, transplant, CV mortality, and HF hospitalization | 2 | 0.87 | 0.76 – 0.99 | 49% | 0.03* |
| All-cause mortality and HF hospitalization | 3 | 0.96 | 0.90 – 1.02 | 88% | 0.20 |
| CV mortality and HF rehospitalization | 4 | 0.92 | 0.81 – 0.94 | 60% | <0.01* |
| Transplant and all-cause mortality | 6 | 0.85 | 0.82 – 0.89 | 64% | <0.01* |
| Transplant and CV mortality | 2 | 0.92 | 0.83 – 1.01 | 78% | 0.08 |
| HF hospitalization | 2 | 0.85 | 0.78 – 0.91 | 66% | <0.01* |
| **Unadjusted** | | | | | |
| Outcome | N of studies | HR | 95% CI | I^2^ | P value |
| All-cause mortality or HF  rehospitalization | 2 | 0.91 | 0.84 | 46% | 0.02* |
| VAD, transplant, CV mortality, and HF hospitalization | - | - | - | - | - |
| All-cause mortality and HF hospitalization | 6 | 0.89 | 0.86 – 0.92 | 29% | <0.01* |
| CV mortality and HF rehospitalization | 3 | 0.84 | 0.76 – 0.93 | 79% | <0.01* |
| Transplant and all-cause mortality | 6 | 0.85 | 0.83 – 0.88 | 29% | <0.01* |
| Transplant and CV mortality | 2 | 0.88 | 0.85 – 0.92 | 0% | <0.01* |
| HF hospitalization | 3 | 0.85 | 0.80 – 0.89 | 73% | <0.01* |

*Indicates significance.
CV, cardiovascular; HF, heart failure; VAD, ventricular assist device
