# Supplementary material for: Prognostic impact of peak oxygen consumption in heart failure: A systematic review and meta‐analysis
Source: ESC Heart Fail. 2025 Aug 12;12(5):3624–42. doi: 10.1002/ehf2.15391 (PMC12450781; doi:10.1002/ehf2.15391)
Supplement: Supplementary file 5 — Table S4. Egger's test and Trim and Fill method. [file EHF2-12-3624-s022.docx]

**Table S4.** Egger’s test and Trim and Fill method.

| **Egger’s Test**  Test for Funnel Plot Asymmetry: z = -3.4278, p = 0.0006  Limit Estimate (as sei -> 0): b = 0.9883 (CI: 0.8901, 1.0865) |
| --- |
| **Trim and Fill**  Number of studies: k = 17 (with 0 added studies)  95%-CI z p-value  Random effects model: 0.8219 [0.7510; 0.8928] 22.72 < 0.0001  Quantifying heterogeneity:  tau^2 = 0.0222 [0.0123; 0.0517]; tau = 0.1491 [0.1111; 0.2273]  I^2 = 100.0%; H = 109.77  Test of heterogeneity:  Q df p-value  192783.46 16 <0.01 |
