# Supplementary material for: Prognostic impact of peak oxygen consumption in heart failure: A systematic review and meta‐analysis
Source: ESC Heart Fail. 2025 Aug 12;12(5):3624–42. doi: 10.1002/ehf2.15391 (PMC12450781; doi:10.1002/ehf2.15391)
Supplement: Supplementary file 6 — Table S5. Meta‐regression analyses based on age, sex, BMI, LVEF, and reported comorbidities. [file EHF2-12-3624-s012.docx]

**Table S4.** Meta-regression analyses based on age, sex, BMI, LVEF, and reported comorbidities.

| **Outcomes** | *r* | SE | 95%CI | *z* | P |
| --- | --- | --- | --- | --- | --- |
| **Age** |  |  |  |  |  |
| All-cause mortality | 0.017 | 0.001 | 0.01 – 0.02 | 12.04 | <0.01* |
| All-cause mortality and HF hospitalization** | - | - | - | - | - |
| Transplant and all-cause mortality | 0.018 | 0.004 | 0.01 – 0.03 | 4.68 | <0.01* |
| HF hospitalization | 0.017 | 0.006 | 0.01 – 0.03 | 2.78 | <0.01* |
| **Sex** |  |  |  |  |  |
| All-cause mortality | 1.225 | 0.068 | 1.09 – 1.36 | 18.08 | <0.01* |
| All-cause mortality and HF hospitalization | 1.948 | 0.184 | 1.59 – 2.31 | 10.57 | <0.01* |
| Transplant and all-cause mortality | 1.358 | 0.103 | 1.16 – 1.56 | 13.16 | <0.01* |
| HF hospitalization | 1.519 | 0.205 | 1.12 – 1.92 | 7.41 | <0.01* |
| **BMI** |  |  |  |  |  |
| All-cause mortality | 0.037 | 0.003 | 0.03 – 0.04 | 11.31 | <0.01* |
| All-cause mortality and HF hospitalization | 0.039 | 0.002 | 0.03 – 0.04 | 16.64 | <0.01* |
| Transplant and all-cause mortality | 0.034 | 0.006 | 0.02 – 0.05 | 5.42 | <0.01* |
| HF hospitalization | 0.036 | 0.022 | -0.01 – 0.08 | 1.63 | 0.10 |
| **LVEF%** |  |  |  |  |  |
| All-cause mortality | 0.030 | 0.001 | 0.03 – 0.03 | 26.25 | <0.01* |
| All-cause mortality and HF hospitalization | 0.028 | 0.002 | 0.02 – 0.03 | 14.52 | <0.01* |
| Transplant and all-cause mortality | 0.029 | 0.002 | 0.02 – 0.03 | 11.72 | <0.01* |
| HF hospitalization | 0.027 | 0.002 | 0.02 – 0.03 | 17.14 | <0.01* |
| **Atrial fibrillation** |  |  |  |  |  |
| All-cause mortality | 5.214 | 1.244 | 2.78 – 7.65 | 4.19 | <0.01* |
| Transplant and all-cause mortality | 7.976 | 0.596 | 6.81 – 9.14 | 13.39 | <0.01* |
| HF hospitalization | 3.137 | 0.592 | 1.98 – 4.30 | 5.30 | <0.01* |
| **Hypertension** |  |  |  |  |  |
| All-cause mortality | 1.431 | 0.128 | 1.18 – 1.68 | 11.15 | <0.01* |
| Transplant and all-cause mortality** | - | - | - | - | - |
| HF hospitalization | 1.99 | 0.261 | 1.48 – 2.50 | 7.62 | <0.01* |
| **Chronic Kidney Disease** |  |  |  |  |  |
| All-cause mortality** | - | - | - | - | - |
| Transplant and all-cause mortality** | - | - | - | - | - |
| HF hospitalization | 4.852 | 0.273 | 4.32 – 5.39 | 17.78 | <0.01* |
| **Diabetes** |  |  |  |  |  |
| All-cause mortality | 3.066 | 0.183 | 2.71 – 3.42 | 16.75 | <0.01* |
| Transplant and all-cause mortality | 3.745 | 0.302 | 3.15 – 4.34 | 12.39 | <0.01* |
| HF hospitalization | 4.127 | 0.365 | 3.41 – 4.84 | 11.31 | <0.01* |
| **ACE-i/ARBs** |  |  |  |  |  |
| All-cause mortality | 0.015 | 0.001 | 0.01 – 0.02 | 24.19 | <0.01* |
| **B-blockers** |  |  |  |  |  |
| All-cause mortality | 0.016 | 0.000 | 0.02 – 0.02 | 34.74 | <0.01* |
| **Diuretics** |  |  |  |  |  |
| All-cause mortality | 0.015 | 0.001 | 0.01 – 0.02 | 29.09 | <0.01* |
| **Statins** |  |  |  |  |  |
| All-cause mortality | 0.023 | 0.002 | 0.02 – 0.03 | 11.90 | <0.01* |

*Indicates significance.
**Insufficient observations.
BMI, body mass index; HF, heart failure; LVEF, left ventricular ejection fraction
