# Supplementary material for: Prognostic impact of peak oxygen consumption in heart failure: A systematic review and meta‐analysis
Source: ESC Heart Fail. 2025 Aug 12;12(5):3624–42. doi: 10.1002/ehf2.15391 (PMC12450781; doi:10.1002/ehf2.15391)
Supplement: Supplementary file 7 — Table S6. Risk of bias and quality assessment of the included studies. [file EHF2-12-3624-s009.docx]

**Table S6.** Risk of bias and quality assessment of the included studies.

|  | | | | | | | | | | | | |  |  |
| --- | --- | --- | --- | --- | --- | --- | --- | --- | --- | --- | --- | --- | --- | --- |
| Author, Year | Selection | | | | | Comparability | | Exposure | | | | Total score | | |
|  | Representativeness of the intervention cohort | Selection of the non-exposed cohort | Ascertainment of exposure | Demonstration that outcome of interest was not present at start of study | Control for important or additional factors | | Assessment of outcome | | Was follow up long enough for outcomes to occur | Adequacy of follow up of cohorts |  | | |  |
| Al-Najjar 2012 | **⋆** | **⋆** | **⋆** | **⋆** |  | |  | | **⋆** | **⋆** | 6 | | |  |
| Baldi 2018 |  | **⋆** | **⋆** | **⋆** |  | | **⋆** | | **⋆** | **⋆** | 6 | | |  |
| Bras 2023 | **⋆** | **⋆** | **⋆** | **⋆** | **⋆** | | **⋆** | | **⋆** | **⋆** | 8 | | |  |
| Shafiq 2016 | **⋆** | **⋆** | **⋆** | **⋆** | **⋆** | | **⋆** | | **⋆** | **⋆** | 8 | | |  |
| Gordon 2023 | **⋆** | **⋆** | **⋆** | **⋆** | **⋆⋆** | | **⋆** | | **⋆** |  | 8 | | |  |
| Goda 2011 | **⋆** | **⋆** | **⋆** | **⋆** | **⋆** | | **⋆** | | **⋆** | **⋆** | 8 | | |  |
| Hsich 2007 | **⋆** | **⋆** | **⋆** | **⋆** | **⋆** | | **⋆** | | **⋆** |  | 7 | | |  |
| Meyer 2001 | **⋆** | **⋆** | **⋆** | **⋆** | **⋆** | | **⋆** | | **⋆** |  | 7 | | |  |
| Myers 2021 | **⋆** | **⋆** |  | **⋆** | **⋆** | |  | | **⋆** |  | 5 | | |  |
| Hsu 2019 | **⋆** | **⋆** | **⋆** | **⋆** | **⋆⋆** | |  | | **⋆** |  | 7 | | |  |
| Gitt 2002 | **⋆** | **⋆** | **⋆** | **⋆** | **⋆⋆** | | **⋆** | | **⋆** | **⋆** | 9 | | |  |
| Hansen 2001 | **⋆** |  | **⋆** | **⋆** |  | |  | | **⋆** |  | 4 | | |  |
| Opasich 2001 | **⋆** | **⋆** | **⋆** | **⋆** |  | |  | | **⋆** |  | 5 | | |  |
| Van Iterson 2021 | **⋆** | **⋆** | **⋆** | **⋆** |  | | **⋆** | | **⋆** |  | 6 | | |  |
| Jankowska 2007 | **⋆** | **⋆** | **⋆** | **⋆** | **⋆** | | **⋆** | | **⋆** | **⋆** | 8 | | |  |
| Nakanishi 2014 | **⋆** | **⋆** | **⋆** | **⋆** | **⋆** | |  | | **⋆** |  | 6 | | |  |
| Saitoh 2016 | **⋆** | **⋆** |  | **⋆** | **⋆** | |  | | **⋆** |  | 5 | | |  |
| Pugliese 2021 | **⋆** | **⋆** | **⋆** | **⋆** | **⋆** | | **⋆** | | **⋆** |  | 7 | | |  |
| Magri 2015 | **⋆** | **⋆** | **⋆** | **⋆** |  | | **⋆** | | **⋆** |  | 6 | | |  |
| Pugliese 2019 | **⋆** | **⋆** | **⋆** | **⋆** | **⋆** | | **⋆** | | **⋆** |  | 7 | | |  |
| Szabo 2011 |  |  | **⋆** | **⋆** |  | |  | | **⋆** |  | 3 | | |  |
| Walsh 1997 | **⋆** |  | **⋆** | **⋆** |  | |  | | **⋆** |  | 4 | | |  |
| Tseliou 2013 |  |  |  | **⋆** |  | |  | | **⋆** |  | 2 | | |  |
| Ehrman 2018 | **⋆** | **⋆** | **⋆** | **⋆** |  | | **⋆** | | **⋆** |  | 6 | | |  |
| Sato 2015 | **⋆** | **⋆** | **⋆** | **⋆** | **⋆⋆** | | **⋆** | | **⋆** |  | 8 | | |  |
| Shibata 2018 | **⋆** | **⋆** | **⋆** | **⋆** |  | | **⋆** | | **⋆** | **⋆** | 7 | | |  |
| Chen 2023 | **⋆** | **⋆** | **⋆** | **⋆** | **⋆** | | **⋆** | | **⋆** |  | 7 | | |  |
| Chen 2024 | **⋆** | **⋆** | **⋆** | **⋆** | **⋆** | | **⋆** | | **⋆** |  | 7 | | |  |
| O'Neill 2005 | **⋆** | **⋆** | **⋆** | **⋆** | **⋆** | |  | | **⋆** |  | 6 | | |  |
| Kallistratos 2008 | **⋆** | **⋆** | **⋆** | **⋆** | **⋆** | |  | | **⋆** | **⋆** | 7 | | |  |
| Li 2024 | **⋆** | **⋆** | **⋆** | **⋆** |  | | **⋆** | | **⋆** | **⋆** | 7 | | |  |
| Woods 2011 | **⋆** |  | **⋆** | **⋆** |  | | **⋆** | | **⋆** |  | 5 | | |  |
| Myers 2009 | **⋆** | **⋆** | **⋆** | **⋆** |  | | **⋆** | | **⋆** |  | 6 | | |  |
| Romuk 2020 | **⋆** | **⋆** |  | **⋆** | **⋆** | | **⋆** | | **⋆** |  | 6 | | |  |
| Lee 2012 | **⋆** |  | **⋆** | **⋆** |  | | **⋆** | | **⋆** |  | 5 | | |  |
| Zhuang 2021 | **⋆** | **⋆** |  | **⋆** |  | | **⋆** | | **⋆** | **⋆** | 6 | | |  |
| Cunha 2023 | **⋆** | **⋆** | **⋆** | **⋆** | **⋆** | | **⋆** | | **⋆** |  | 7 | | |  |
| Eslam 2022 | **⋆** | **⋆** | **⋆** | **⋆** |  | | **⋆** | |  |  | 5 | | |  |
| Koerber 2019 |  | **⋆** | **⋆** | **⋆** |  | |  | | **⋆** |  | 4 | | |  |
| Nakanishi 2022 | **⋆** | **⋆** | **⋆** | **⋆** | **⋆⋆** | |  | | **⋆** |  | 7 | | |  |
| Silverii 2023 | **⋆** | **⋆** | **⋆** | **⋆** | **⋆** | | **⋆** | | **⋆** |  | 7 | | |  |
| Murata 2019 | **⋆** | **⋆** | **⋆** | **⋆** | **⋆** | |  | | **⋆** |  | 6 | | |  |
| Corra 2020 | **⋆** | **⋆** | **⋆** | **⋆** |  | | **⋆** | | **⋆** |  | 6 | | |  |
| Hoyer 2008 | **⋆** | **⋆** |  | **⋆** | **⋆** | | **⋆** | | **⋆** |  | 6 | | |  |
| Magri 2020 | **⋆** | **⋆** | **⋆** | **⋆** | **⋆** | |  | | **⋆** |  | 6 | | |  |
| Malhotra 2016 | **⋆** | **⋆** | **⋆** | **⋆** | **⋆⋆** | | **⋆** | | **⋆** | **⋆** | 9 | | |  |
| Shen 2016 | **⋆** | **⋆** | **⋆** | **⋆** | **⋆⋆** | | **⋆** | | **⋆** | **⋆** | 9 | | |  |
| de Groote 2004 | **⋆** | **⋆** |  | **⋆** | **⋆** | |  | | **⋆** | **⋆** | 6 | | |  |
| Lamblin 2005 | **⋆** |  |  | **⋆** | **⋆⋆** | | **⋆** | | **⋆** | **⋆** | 7 | | |  |
| Guazzi 2009 | **⋆** | **⋆** | **⋆** | **⋆** |  | | **⋆** | | **⋆** |  | 6 | | |  |
| Jorde 2007 | **⋆** | **⋆** | **⋆** | **⋆** | **⋆⋆** | | **⋆** | | **⋆** | **⋆** | 9 | | |  |
| Lala 2021 |  | **⋆** | **⋆** | **⋆** | **⋆⋆** | | **⋆** | | **⋆** |  | 7 | | |  |
| Nadruz 2017 | **⋆** | **⋆** | **⋆** | **⋆** | **⋆⋆** | | **⋆** | | **⋆** | **⋆** | 9 | | |  |
| Vecchiato 2024 | **⋆** | **⋆** | **⋆** | **⋆** | **⋆⋆** | |  | | **⋆** |  | 7 | | |  |
| Stolker 2006 | **⋆** | **⋆** | **⋆** | **⋆** |  | |  | | **⋆** |  | 5 | | |  |
| Yan 2013 | **⋆** | **⋆** | **⋆** | **⋆** | **⋆** | | **⋆** | | **⋆** | **⋆** | 8 | | |  |
| Czubaszewski 2018 |  | **⋆** | **⋆** | **⋆** |  | |  | | **⋆** |  | 4 | | |  |
| Doehner 2005 |  | **⋆** | **⋆** | **⋆** | **⋆** | | **⋆** | | **⋆** | **⋆** | 7 | | |  |
| Ingle 2012 | **⋆** | **⋆** | **⋆** | **⋆** | **⋆⋆** | |  | | **⋆** |  | 7 | | |  |
| Koike 2000 | **⋆** | **⋆** | **⋆** | **⋆** |  | |  | | **⋆** |  | 5 | | |  |
| Piepoli 2016 | **⋆** | **⋆** | **⋆** | **⋆** | **⋆⋆** | | **⋆** | | **⋆** |  | 8 | | |  |
| Scardovi 2012 | **⋆** | **⋆** |  | **⋆** |  | | **⋆** | | **⋆** | **⋆** | 6 | | |  |
