# Supplementary figures and images for: Prognostic impact of peak oxygen consumption in heart failure: A systematic review and meta‐analysis
Source: ESC Heart Fail. 2025 Aug 12;12(5):3624–42. doi: 10.1002/ehf2.15391 (PMC12450781; doi:10.1002/ehf2.15391)

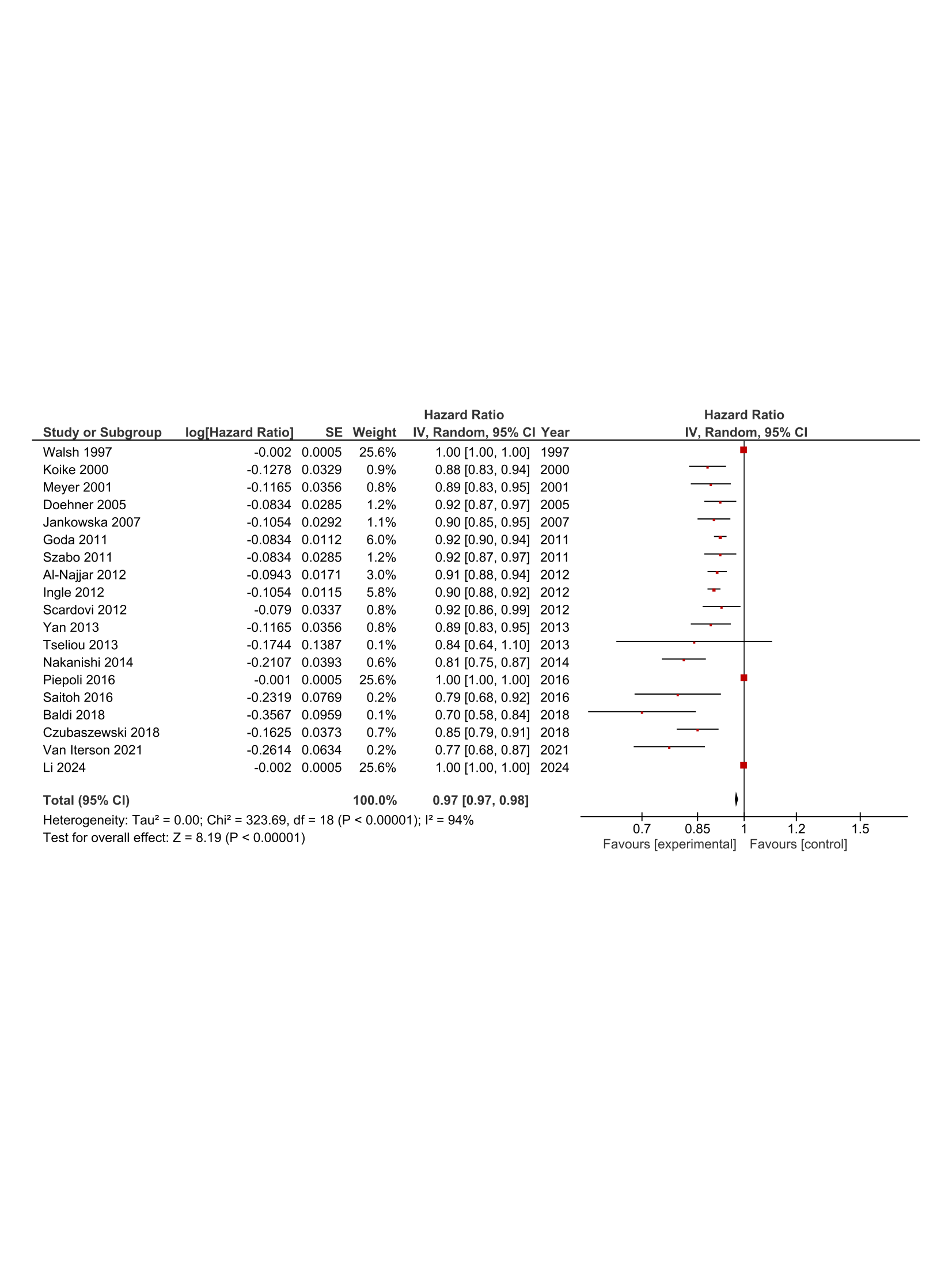

Supplement: Supplementary file 8 — Figure S1. Unadjusted impact of VO2peak on all‐cause mortality per 1 ml/kg/min increase in VO2peak. [file EHF2-12-3624-s013.tiff]

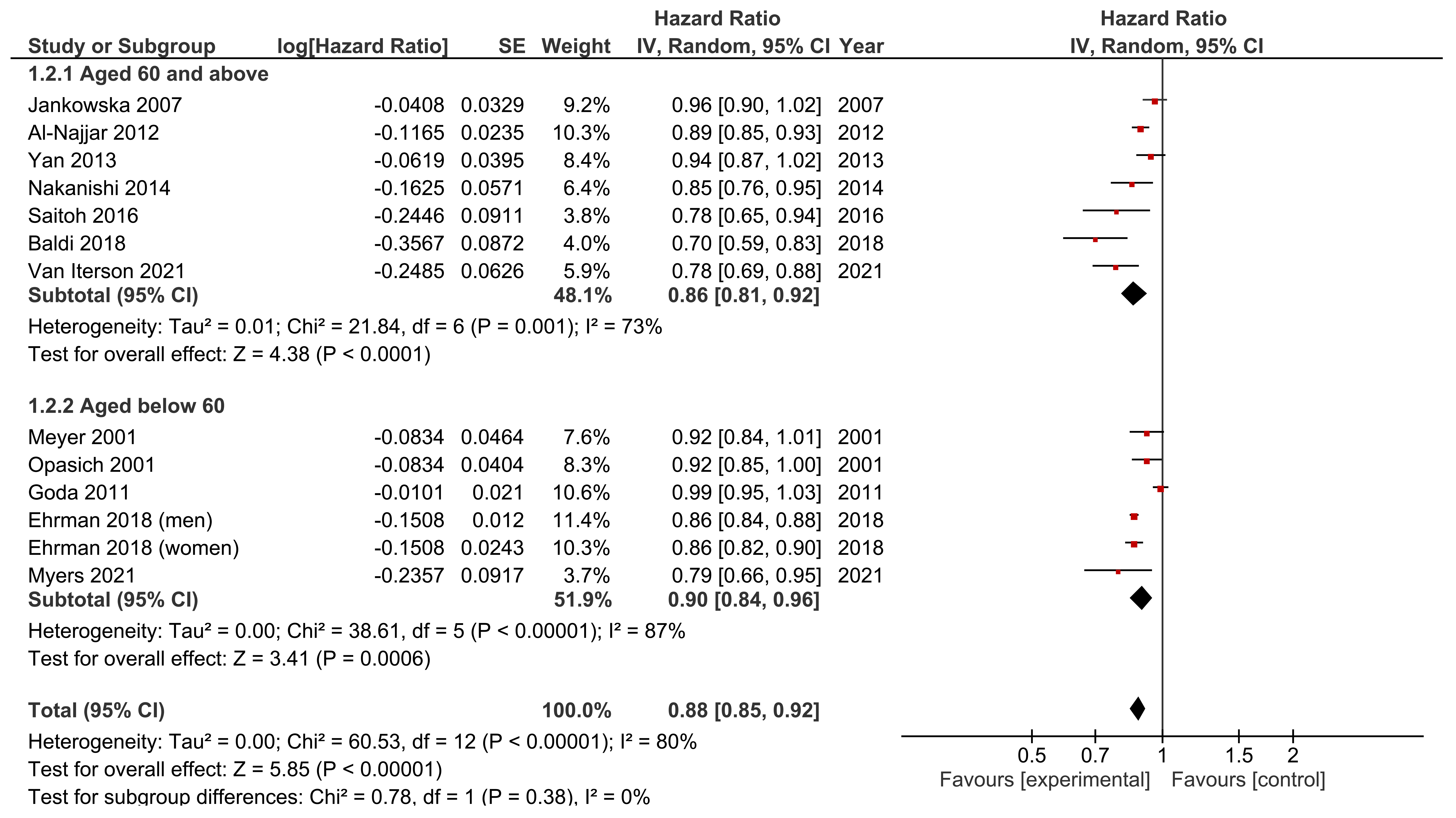

Supplement: Supplementary file 9 — Figure S2. Adjusted impact of VO2peak on all‐cause mortality per 1 ml/kg/min increase in VO2peak, stratified by age. [file EHF2-12-3624-s010.tiff]

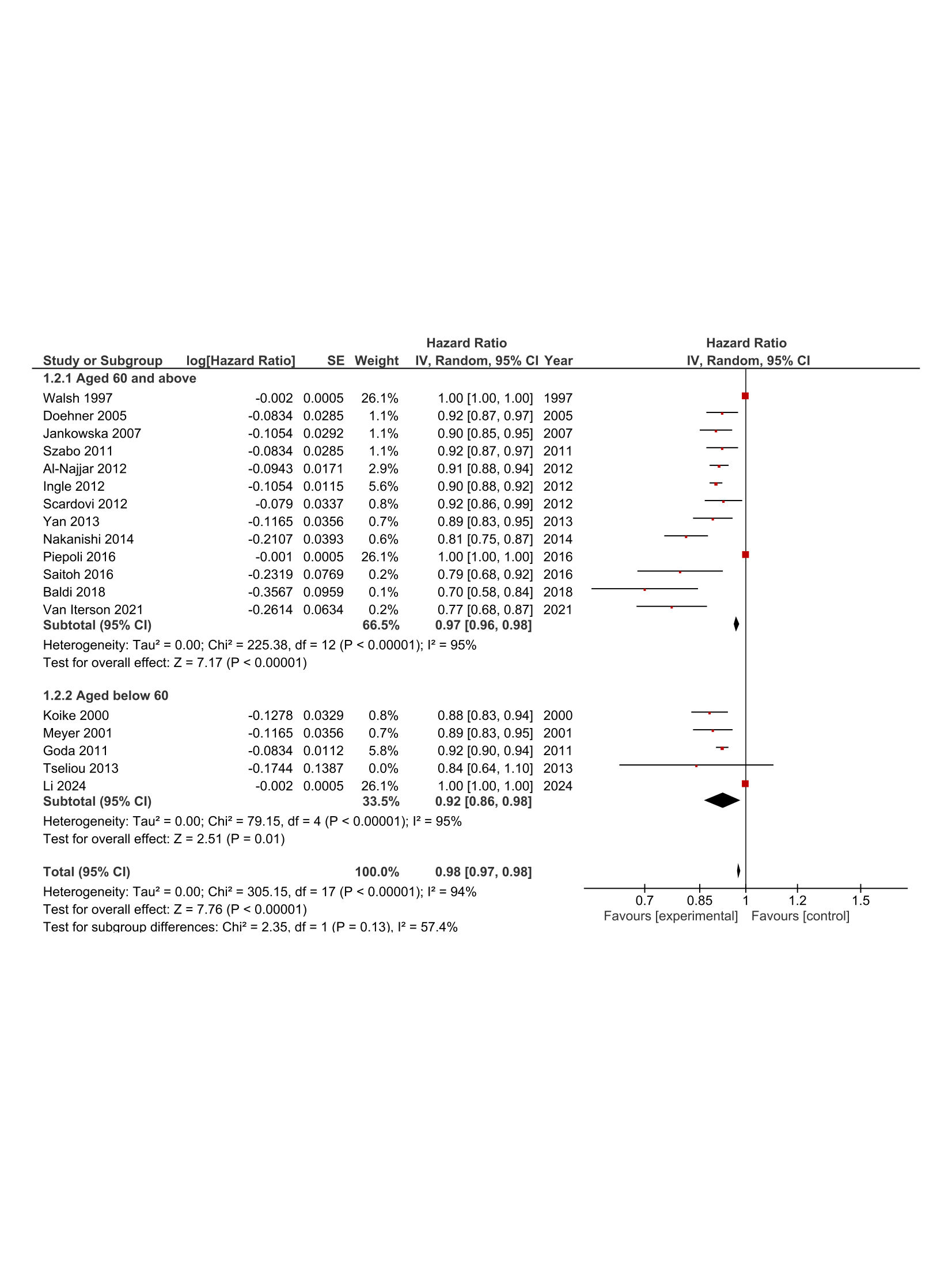

Supplement: Supplementary file 10 — Figure S3. Unadjusted impact of VO2peak on all‐cause mortality per 1 ml/kg/min increase in VO2peak, stratified by age. [file EHF2-12-3624-s005.tiff]

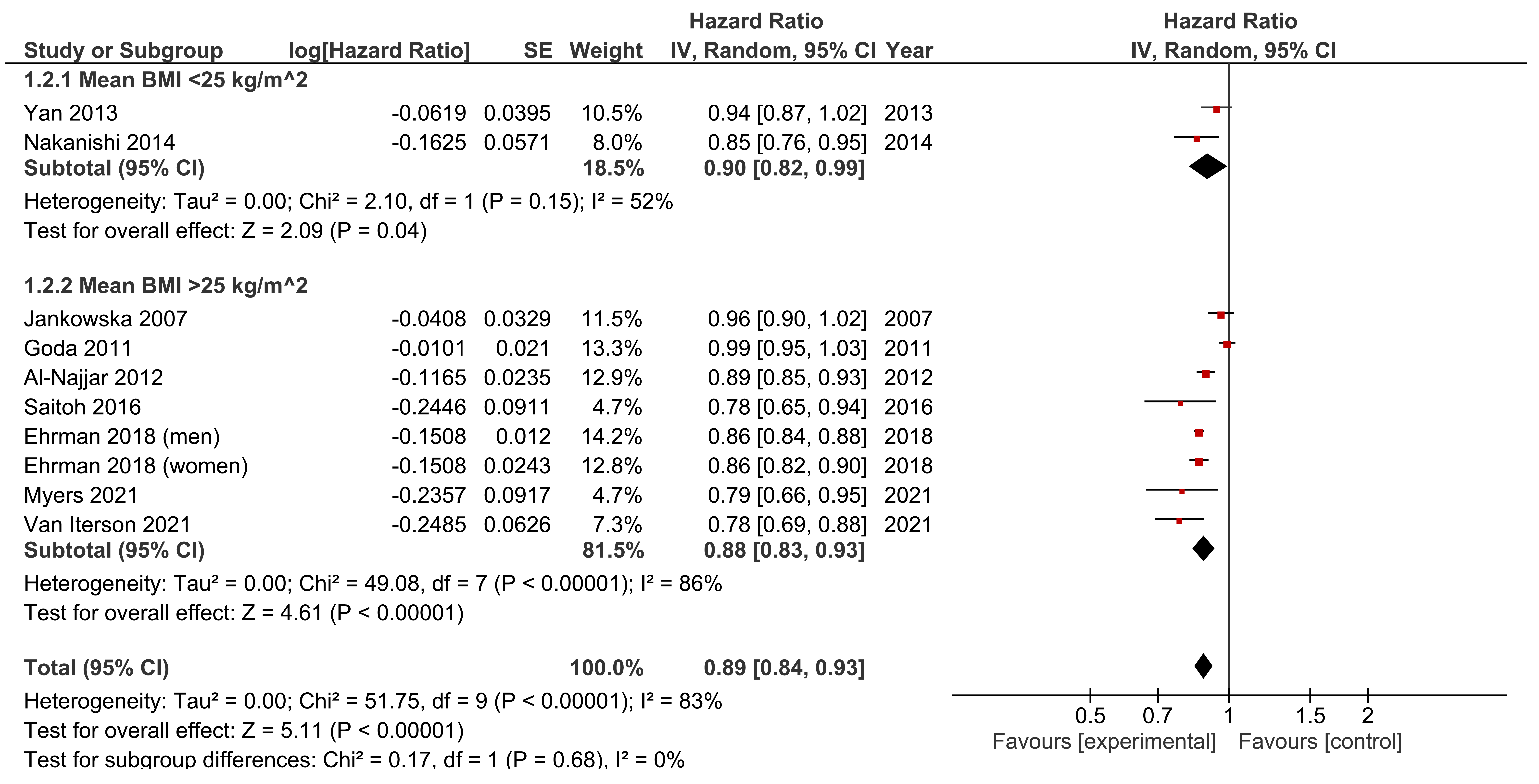

Supplement: Supplementary file 11 — Figure S4. Adjusted impact of VO2peak on all‐cause mortality per 1 ml/kg/min increase in VO2peak, stratified by BMI. [file EHF2-12-3624-s025.tiff]

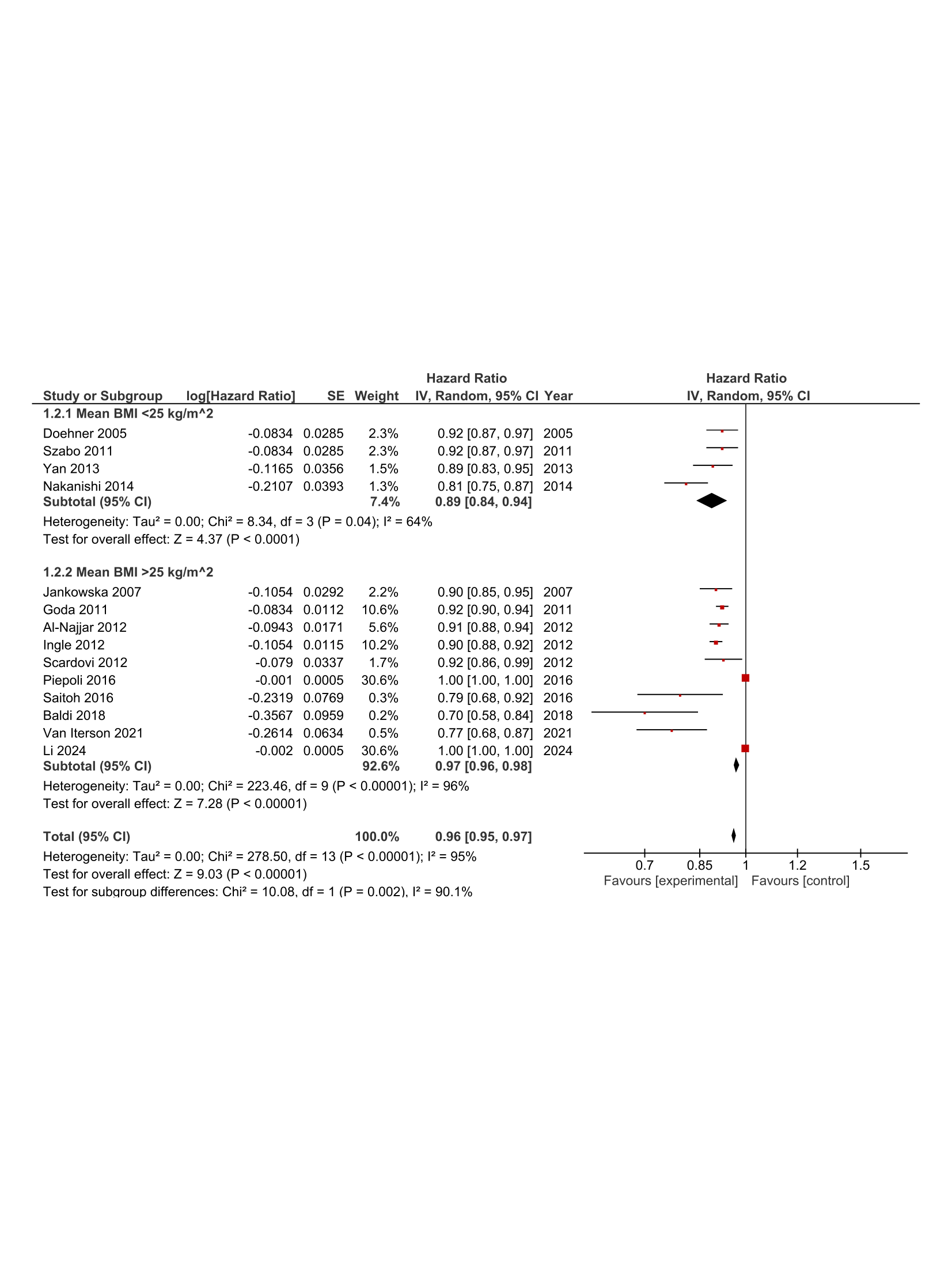

Supplement: Supplementary file 12 — Figure S5. Unadjusted impact of VO2peak on all‐cause mortality per 1 ml/kg/min increase in VO2peak, stratified by BMI. [file EHF2-12-3624-s003.tiff]

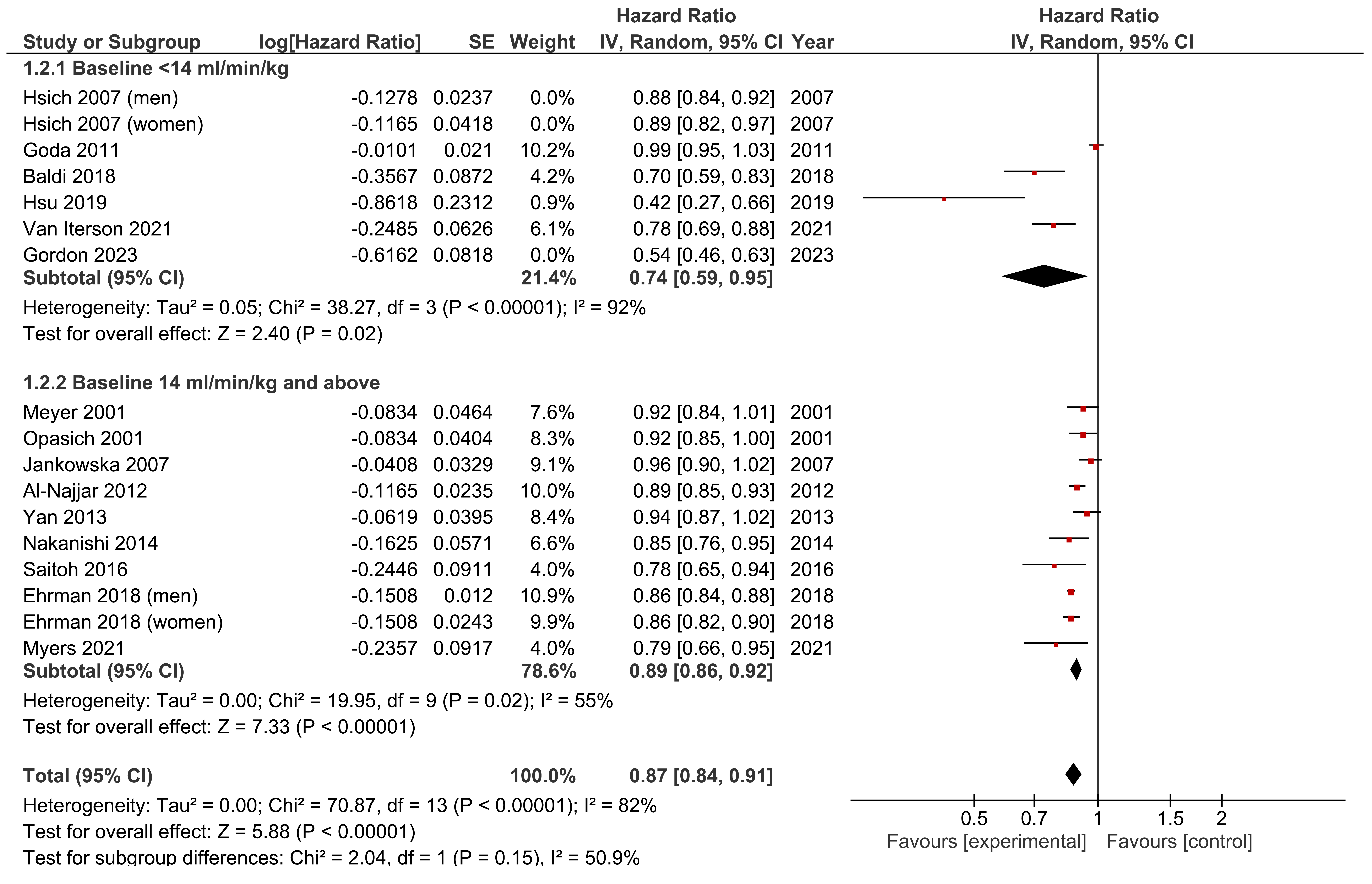

Supplement: Supplementary file 13 — Figure S6. Adjusted impact of VO2peak on all‐cause mortality per 1 ml/kg/min increase in VO2peak, stratified by baseline VO2peak levels. [file EHF2-12-3624-s019.tiff]

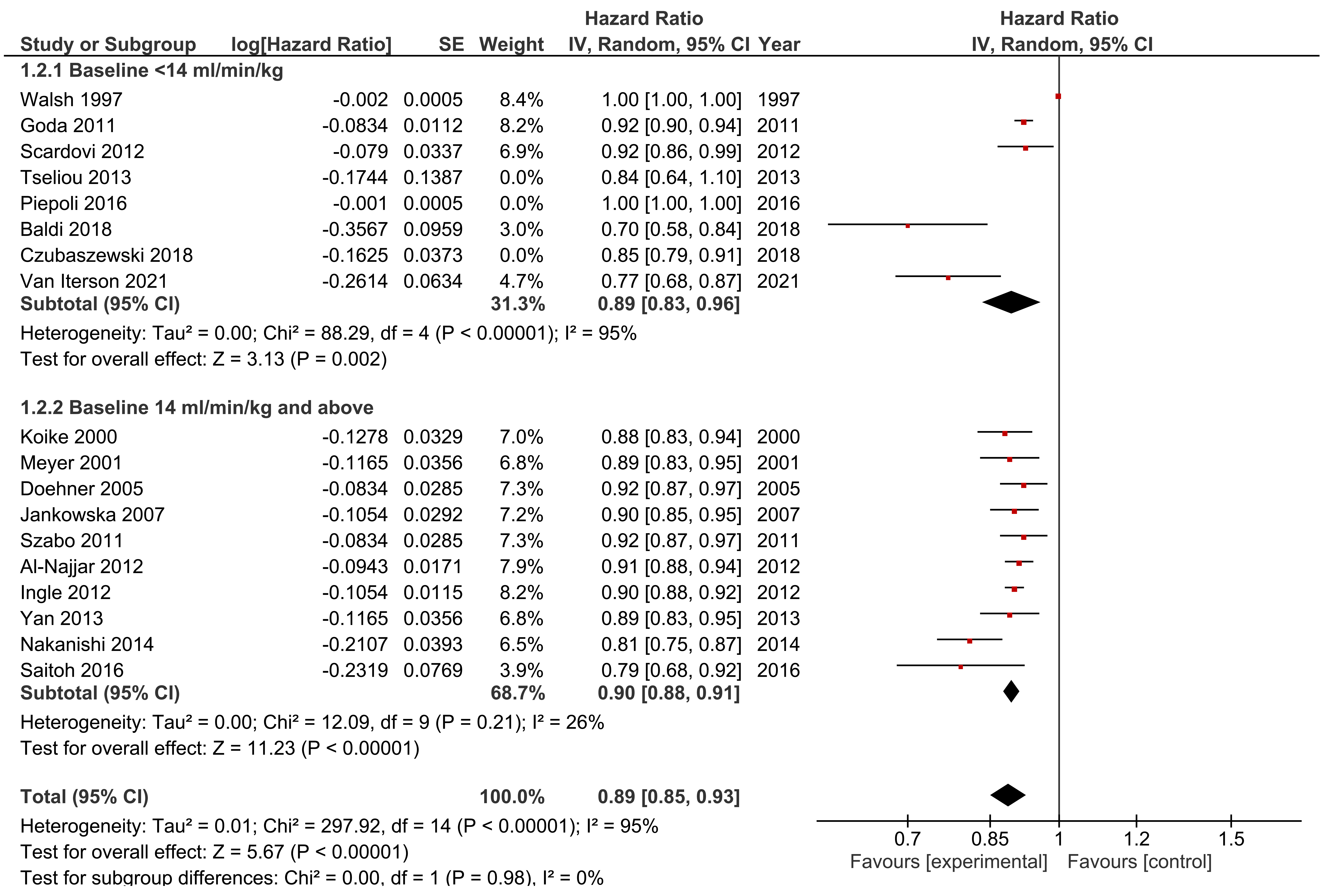

Supplement: Supplementary file 14 — Figure S7. Unadjusted impact of VO2peak on all‐cause mortality per 1 ml/kg/min increase in VO2peak, stratified by baseline VO2peak levels. [file EHF2-12-3624-s017.tiff]

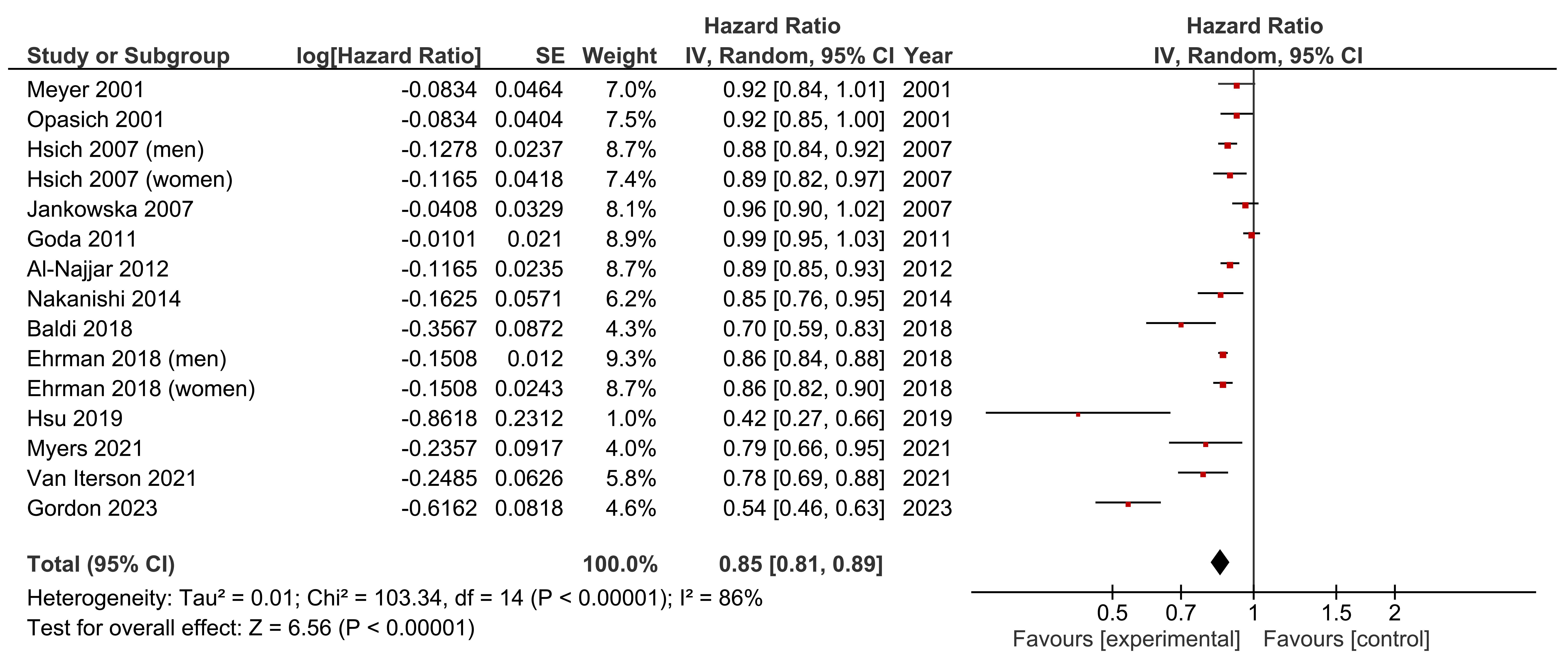

Supplement: Supplementary file 15 — Figure S8. Adjusted impact of VO2peak on all‐cause mortality per 1 ml/kg/min increase in VO2peak among participants with LVEF ≤ 40%. [file EHF2-12-3624-s001.tiff]

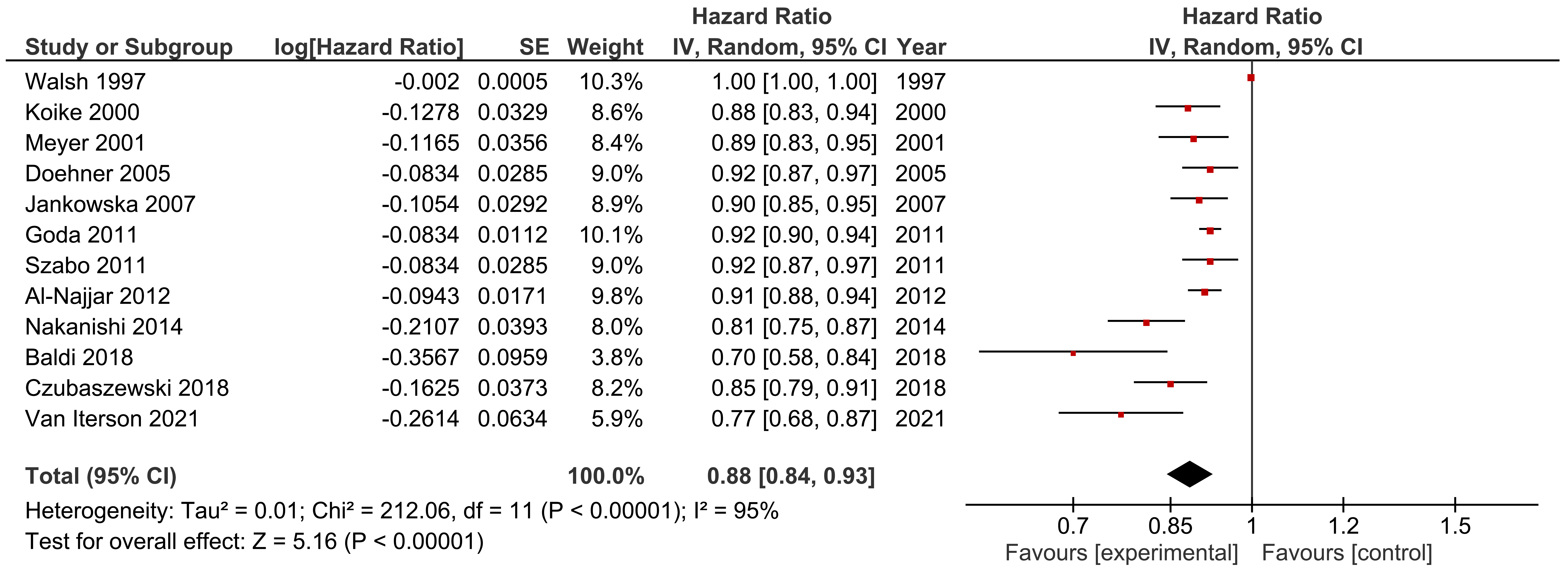

Supplement: Supplementary file 16 — Figure S9. Unadjusted impact of VO2peak on all‐cause mortality per 1 ml/kg/min increase in VO2peak among participants with LVEF ≤ 40%. [file EHF2-12-3624-s007.tiff]

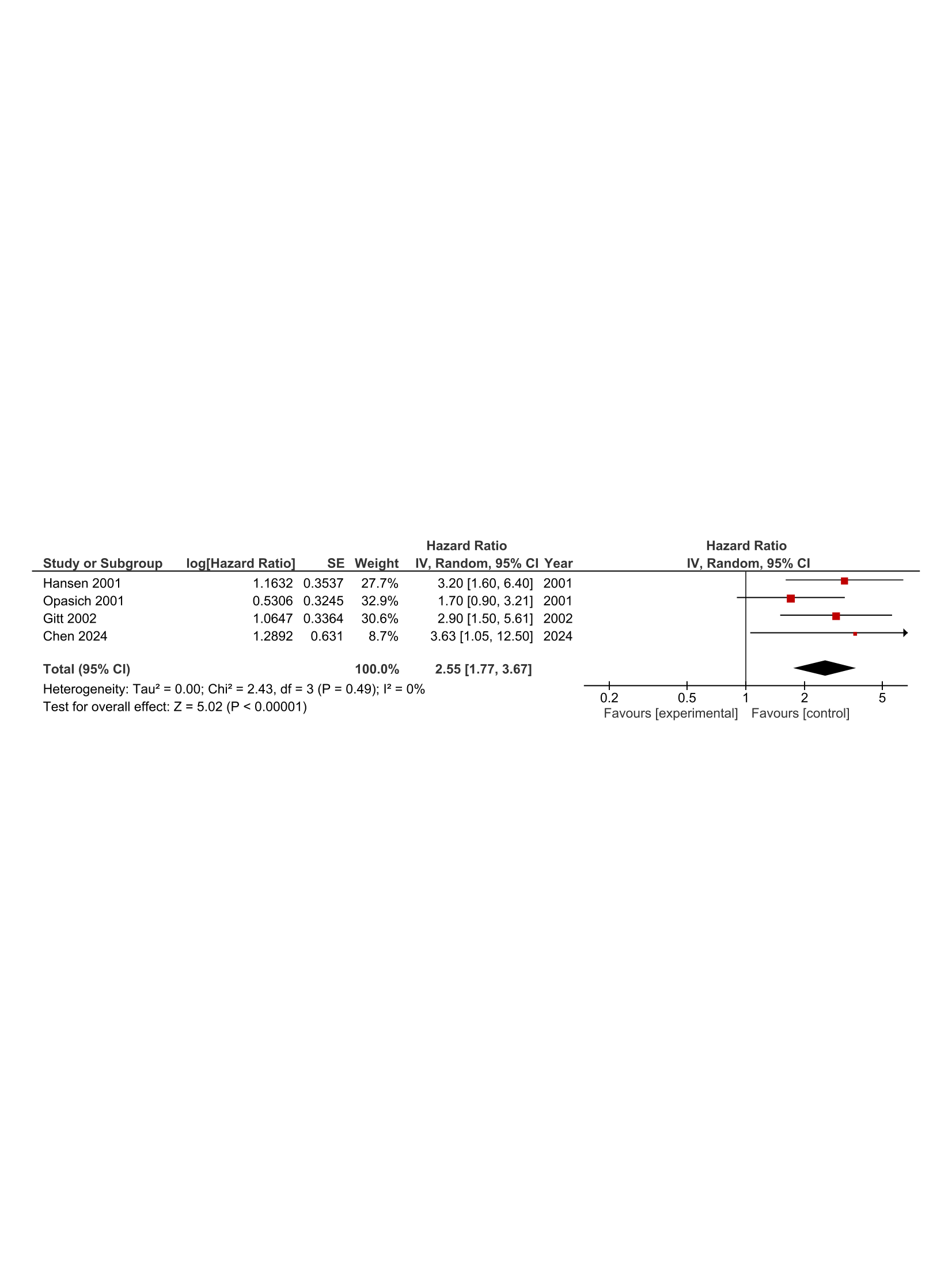

Supplement: Supplementary file 18 — Figure S11. Risk of all‐cause mortality associated with VO2peak equal or below 14 ml/kg/min. [file EHF2-12-3624-s024.tiff]

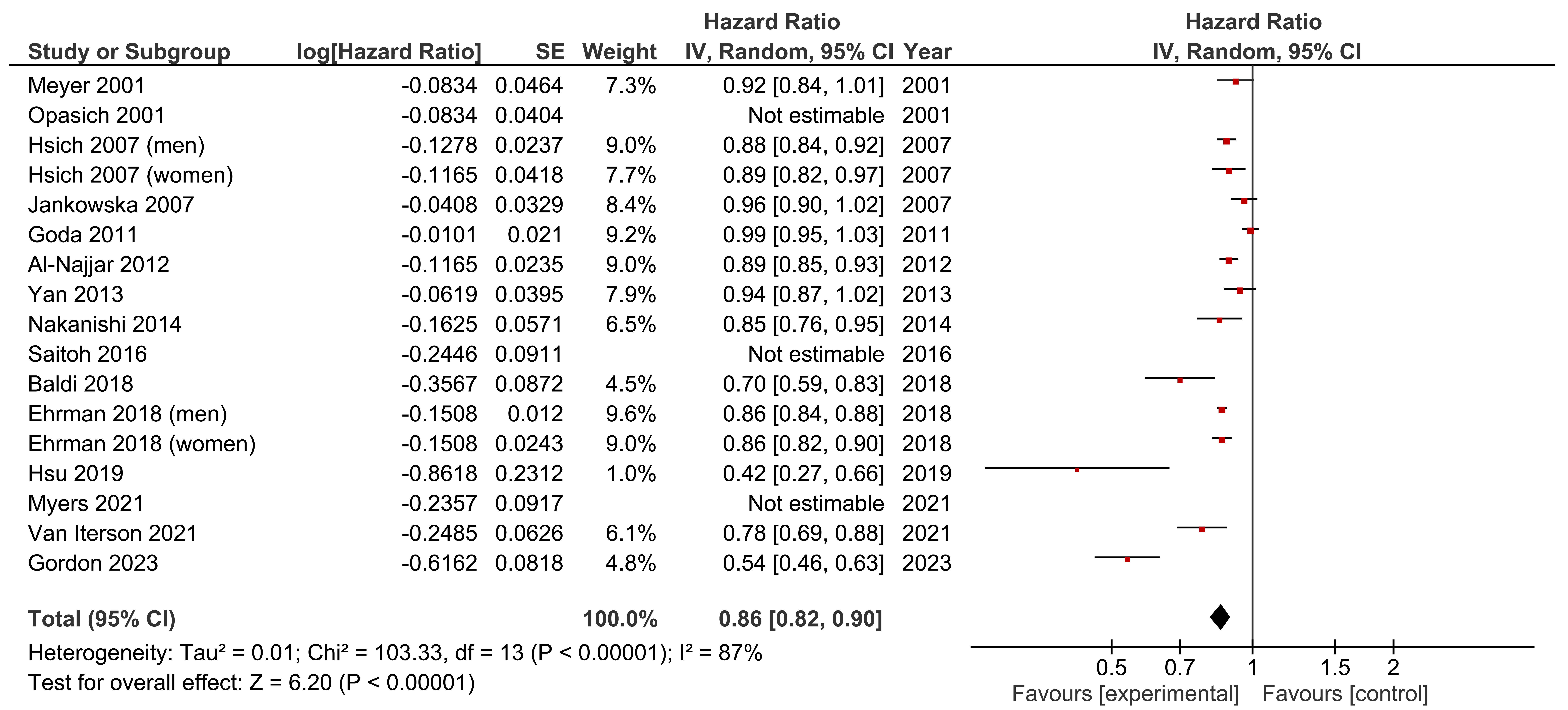

Supplement: Supplementary file 19 — Figure S12. Impact of VO2peak on all‐cause mortality per 1 ml/kg/min increase in VO2peak, with exclusion of high‐risk of bias studies. [file EHF2-12-3624-s018.tiff]

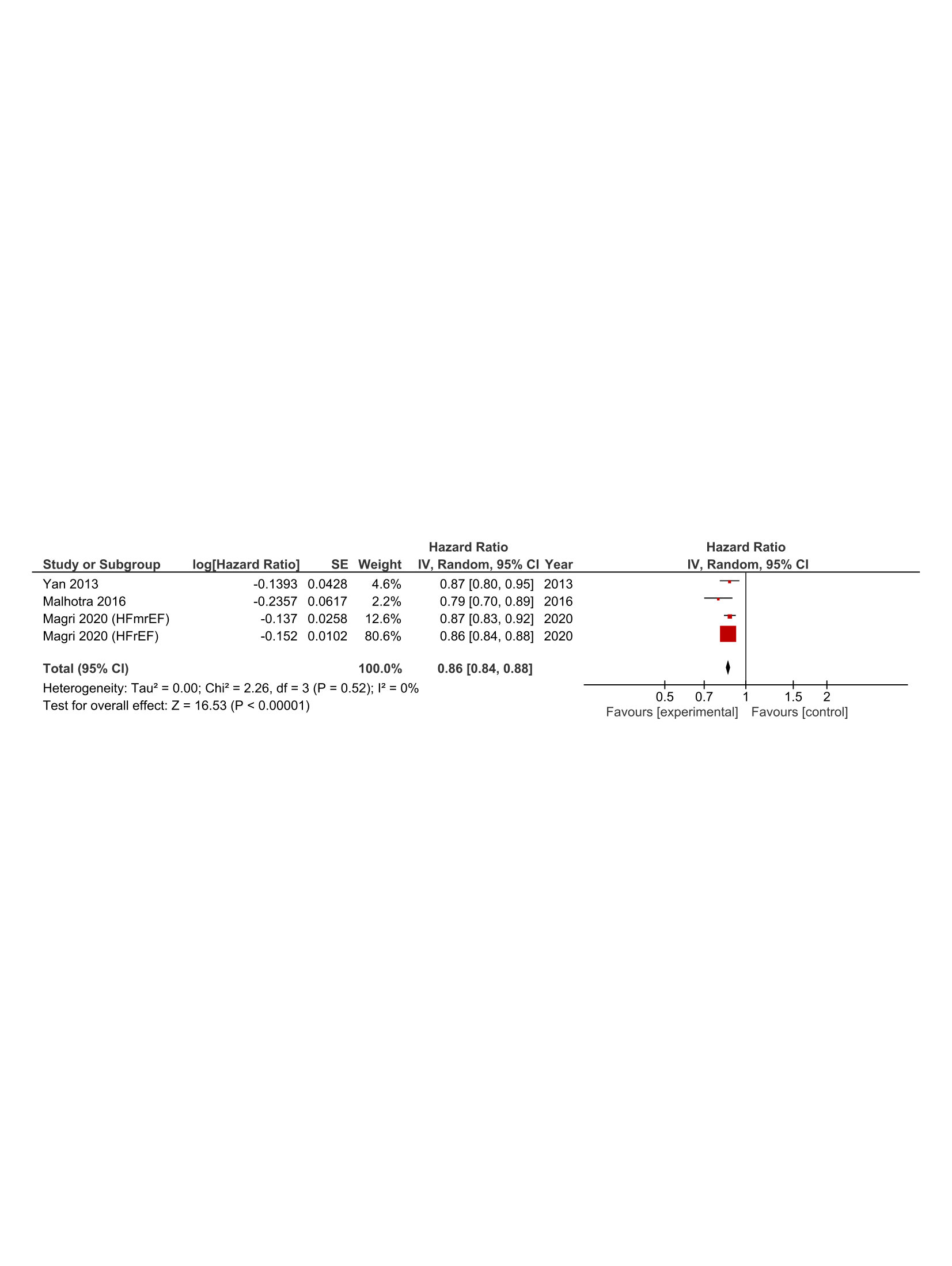

Supplement: Supplementary file 20 — Figure S13. Unadjusted impact of VO2peak on cardiovascular mortality per 1 ml/kg/min increase in VO2peak. [file EHF2-12-3624-s002.tiff]

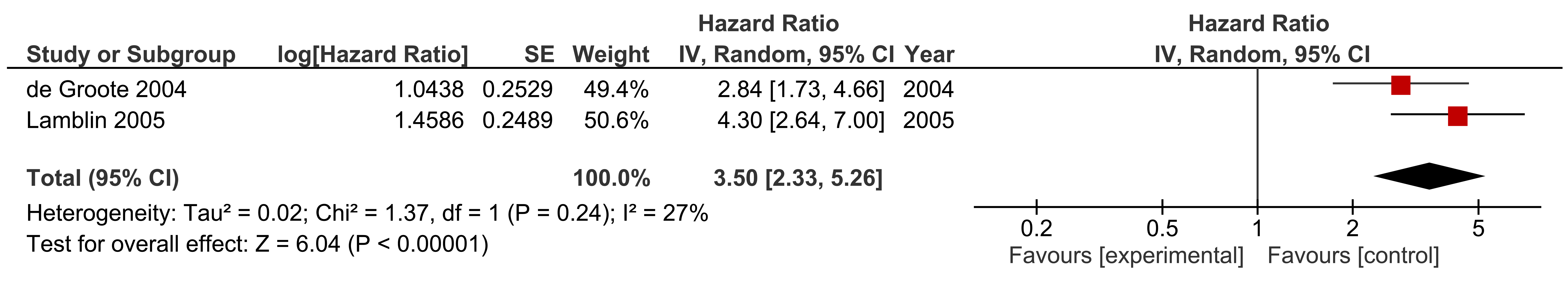

Supplement: Supplementary file 21 — Figure S14. Risk of cardiovascular mortality associated with VO2peak below 50% of predicted value. [file EHF2-12-3624-s008.tiff]

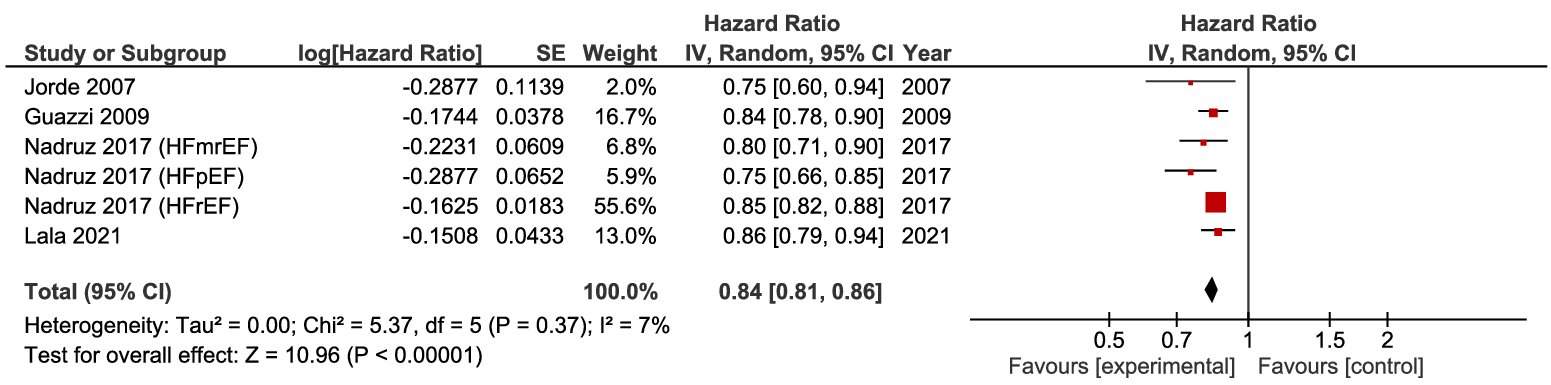

Supplement: Supplementary file 22 — Figure S15. Unadjusted impact of VO2peak on incident ventricular assist device implantation, heart transplant, and all‐cause mortality per 1 ml/kg/min increase in VO2peak. [file EHF2-12-3624-s021.tiff]

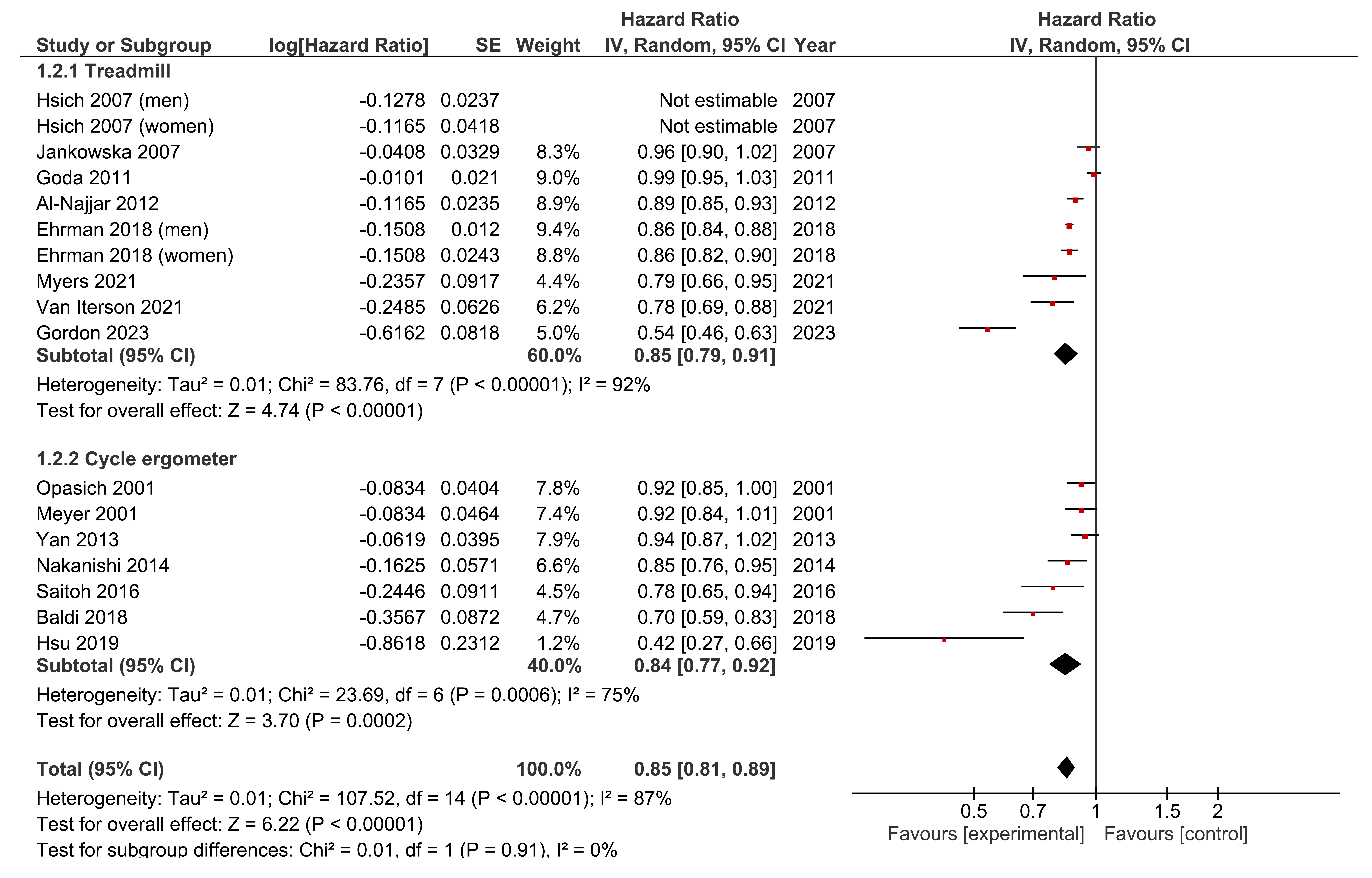

Supplement: Supplementary file 23 — Figure S16. Adjusted impact of VO2peak on all‐cause mortality based on studies using treadmill vs. cycle ergometer. [file EHF2-12-3624-s004.tiff]

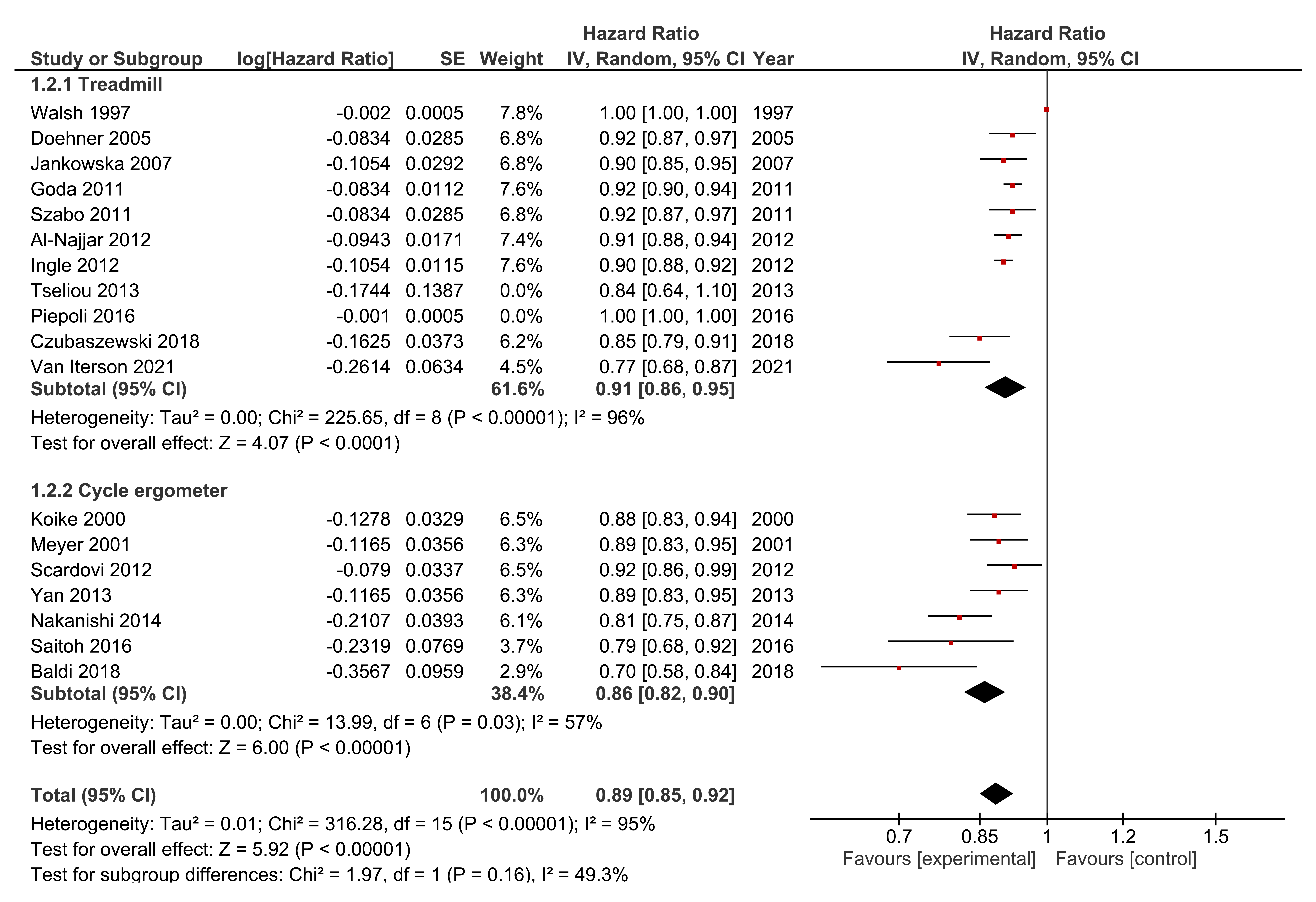

Supplement: Supplementary file 24 — Figure S17. Unadjusted impact of VO2peak on all‐cause mortality based on studies using treadmill vs. cycle ergometer. [file EHF2-12-3624-s026.tiff]

Effect estimate

0  
0.025  
0.05  
0.075  
0.099

0.4

0.6

0.8

1

Effect size

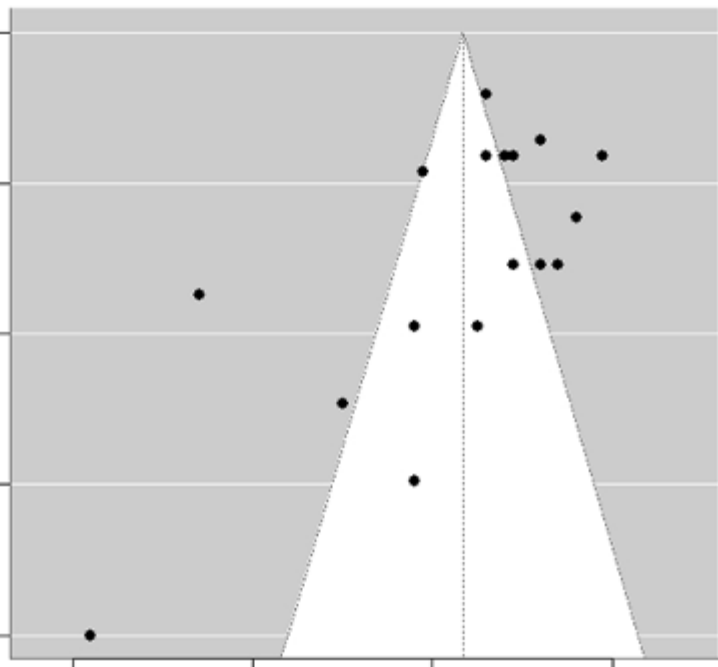

Supplement: Supplementary file 25 — Figure S18. Funnel plot of the included studies. [file EHF2-12-3624-s023.pdf]

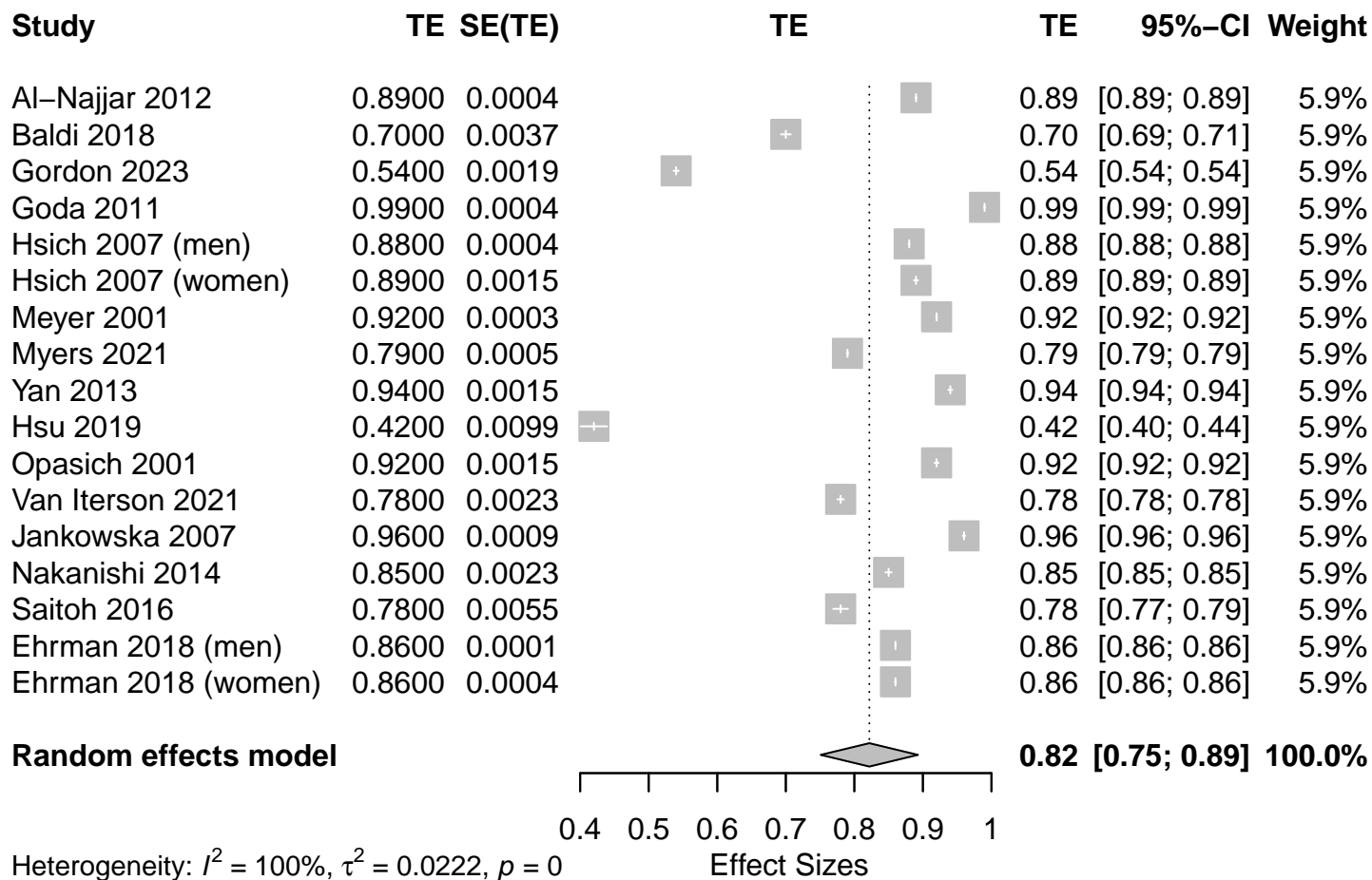

Supplement: Supplementary file 26 — Figure S19. Assessment of publication bias using the Trim and Fill method. [file EHF2-12-3624-s016.pdf]
